# Spectral deep learning for prediction and prospective validation of functional groups†

**DOI:** 10.1039/c9sc06240h

**Published:** 2020-03-13

**Authors:** Jonathan A. Fine, Anand A. Rajasekar, Krupal P. Jethava, Gaurav Chopra

**Affiliations:** Department of Chemistry, Purdue University 560 Oval Drive West Lafayette IN 47907 USA gchopra@purdue.edu; Department of Biological Engineering, Bhupat and Jyoti Mehta School of Biosciences, Indian Institute of Technology Madras Chennai 600036 India

## Abstract

State-of-the-art identification of the functional groups present in an unknown chemical entity requires the expertise of a skilled spectroscopist to analyse and interpret Fourier transform infra-red (FTIR), mass spectroscopy (MS) and/or nuclear magnetic resonance (NMR) data. This process can be time-consuming and error-prone, especially for complex chemical entities that are poorly characterised in the literature, or inefficient to use with synthetic robots producing molecules at an accelerated rate. Herein, we introduce a fast, multi-label deep neural network for accurately identifying all the functional groups of unknown compounds using a combination of FTIR and MS spectra. We do not use any database, pre-established rules, procedures, or peak-matching methods. Our trained neural network reveals patterns typically used by human chemists to identify standard groups. Finally, we experimentally validated our neural network, trained on single compounds, to predict functional groups in compound mixtures. Our methodology showcases practical utility for future use in autonomous analytical detection.

## Abstract 2

A new multi-label deep neural network architecture is used to combine Infrared and mass spectra, trained on single compounds to predict functional groups, and experimentally validated on complex mixtures.

## Graphical abstract

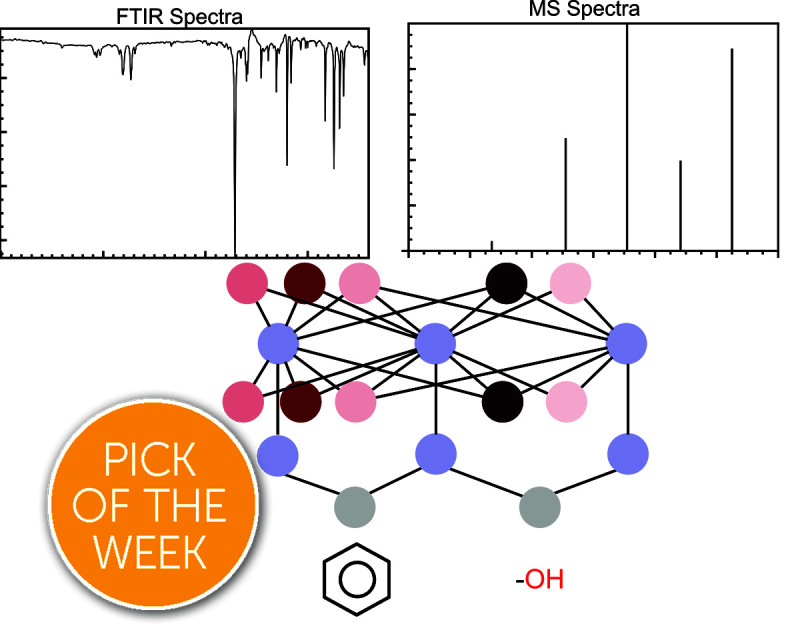

## Introduction

The arrangement of atoms within a molecule dictates its physical, chemical, and spectral properties. Small discrete, or large repeating arrangements of atoms which give rise to measurable changes in a molecule's reactivity,^1–3^ boiling point,^4,5^ melting point,^6,7^ and other characteristics are called functional groups. Given the structural formula of a molecule, a chemist can identify functional groups present (*e.g.* aldehyde, carboxylic acid, alcohol, *etc.*) and can postulate characteristic reactivity and physical properties for a given molecule based on the presence of these groups. Therefore, the identification of functional groups present within an unknown compound is a key step in qualitative organic synthesis and structure elucidation; it is routinely practiced by chemists to validate the synthesis of novel small molecules or identify unknown structures in complex mixtures. Techniques for assigning functional groups based on ‘rules of thumb’ or by matching profiles from known databases are commonly applied in organic chemistry,^8^ metabolomics,^9,10^ and forensic sciences.^11–13^ Furthermore, monitoring of functional group changes can be used to determine the progress of a reaction,^14^ and can even be used to identify the components of complex mixtures for a reaction coordinate.

Chemists often rely on spectroscopic techniques like Fourier transform infrared (FTIR) spectroscopy, mass spectroscopy (MS), and nuclear magnetic resonance (NMR) spectroscopy for the assignment of functional groups. FTIR spectroscopy utilises the frequencies associated with the bonds in a molecule, which typically vibrate around 4000 cm^−1^ to 400 cm^−1^, known as the Infrared region of the electromagnetic spectrum.^8^ This region is associated with specific frequencies that change the oscillating patterns of chemical bonds in the analyte, resulting in an FTIR spectrum.^15^ Typically, a spectroscopist manually analyses this spectrum to identify patterns corresponding to a given functional group using previously established rules and principals,^8^ a time-consuming process subject to human bias and interpretation. Alternatively, if the compound has previously been characterised, the spectroscopist can use software to match the peaks of the analyte to a database of known compounds for identification.^16^

Mass spectroscopy (MS) is another technique commonly used by chemists for the identification of unknown compounds.^8^ One of the first, and still a popular MS ionisation technique, is electron ionisation (EI-MS),^17^ a method performed by bombarding the analyte in the gas phase with high energy electrons (∼70 eV) for molecular ionisation. The resulting cationic radicals are energetically unstable and break apart, resulting in smaller charged particle fragments that are specific to the analyte. Such fragmentation patterns are dependent on molecular functional groups and their arrangements with other functional groups and motifs. The abundance of fragments with a given mass to charge ratio (*m*/*z*) is recorded and reported as the mass spectrum. These spectra are used to search through a database of MS peaks of known compounds, but large-scale automated identification of unknown molecules is still a major challenge.^9,18–20^ In addition, a popular tandem mass spectrometry (MS/MS) method, namely collision-activated dissociation (CAD), has been extensively used for the characterisation of complex mixtures.^21,22^ For CAD, the analyte ions are accelerated and allowed to collide with an inert gas for fragmentation and subsequent MS/MS analysis. Furthermore, in addition to EI-MS and CAD based fragmentation, soft ionisation techniques such as electrospray ionisation mass spectrometry (ESI-MS) have been developed. For ESI-MS, the analyte is sprayed through a spray needle into a carrier gas chamber where an electric field is applied to charge the analyte. This is then passed into a heated capillary which desolvates the analyte, forcing it into the gas phase. Since ESI-MS is a soft ionisation method, it is possible to perform repeated charging of the analyte with no fragmentation due to ionisation. With repeated charging, the (*m*/*z*) values of the resulting ions become lower and detectable. This has been used to determine biomolecular structures, atomic interactions, post-translational modifications, and protein sequence information and has been extended to inorganic, organic, and metal–organic complexes.^23^ However, high-performance liquid chromatography is typically used for molecular fractionation prior to mass-spectrometric analysis to identify the structure of unknown constituents in complex sample mixtures.^23^

Human intervention to analyse the FTIR or MS spectrum is useful but achieving the next generation of autonomous instrumentation for reaction screening requires a completely automated method for determining whether a reaction occurred. The current approaches to automating functional group identification are similar to those applied by humans, using a set of rules and pattern (peak) matching to map spectra to a functional group.^19,24^ Such methods typically utilise only selected spectral regions to identify functional groups, and often afford relatively low confidence predictions owing to a limited database of known compounds.^18^ Furthermore, to our knowledge, these methods can only incorporate data from a single spectral technique (*i.e.*, either FTIR or MS) and ignore relationships between different spectral data for identification. Hence, there is a need for automated and accurate methods capable of multiple-spectra integration without the use of pre-established patterns on known databases. Such methods will need minimal-to-no human intervention, progressing chemistry towards the realisation of automated synthetic robots that screen functional groups and combine spectral data to validate each step during reaction screening and multi-step automated synthesis.^25^ The state-of-the-art robot for automated reaction detection currently employs different techniques to determine the occurrence of a reaction,^26^ but only predefined compounds can be identified. It is a major challenge to develop fully automated robots to discover new reactions that produce unexpected products. Our goal is to extend the capabilities of these automated synthetic robots by developing a fast, automated methodology for functional group determination that can be used in real-time, thereby enabling reaction screening through the identification of functional group changes in a database-free manner.

**Fig. 1:**
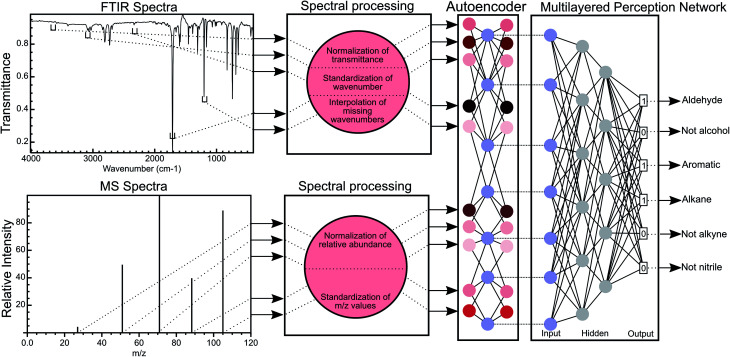
Overview of the MLP methodology for the classification of functional groups using FTIR and MS data. FTIR spectra are processed to normalise the transmittance of the spectra and discretise the wavenumber numbers (creating wavenumber bins), thereby standardising the wavenumbers for all FTIR spectra. Missing wavenumber bins in each spectrum are interpolated using B-splines. A similar process is used for mass spectral data with the exception that no interpolation is performed. The normalised transmittance in all bins is encoded into a latent space by an autoencoder network and this latent space this then used to predict the functional group of a molecule.

Machine learning (ML) is a set of techniques used by computers to perform a specific task without an explicit set of instructions provided by the user. ML techniques have been successfully applied to multiple chemical problems in recent years and still show promise for the advancement of several areas of chemistry. Popular machine learning architectures, such as random forest,^27–29^ multiple layer perception,^30–32^ generalised adversarial networks,^33–37^ and recurrent neural networks,^38–40^ have been used on chemical data for small-molecule design,^41,42^ metabolism,^43,44^ toxicology,^43,45^ photo-electric properties, solubility, and retrosynthesis.^38,40^ It has been shown that the direct molecule as a subgraph of groups of atoms (*i.e.*, functional groups) has distinct advantages over fingerprinting methods.^46,47^ The representation of a molecule or dataset can be reduced to a lower-dimensional latent space by using an autoencoder.^42^ Here, we also used an encoder to create a corresponding latent space based on spectra to predict functional groups which may also be useful to design molecules for specific spectral properties. A few ML techniques to analyse spectra have been used previously^48–52^ but such attempts for functional group prediction used only one type of spectral data; the training data were specific to the application and classified groups separately as a multiple binary classification problem.^51,52^ Binary classifiers are not optimal for a large number of classes and are sensitive to class imbalances during training resulting in problems in identifying all functional groups in a molecule or mixtures.^44,53^ In this work, we present the first ML method, to our knowledge, that integrates FTIR and MS data to obtain a combined set of features as a multi-class, multi-label classification methodology. Our method predicts multiple functional groups for a given molecule in a database-free manner, as compared to identifying a molecule through peak matching or only identifying the major functional group in the molecule (Fig. 1). In this work, we also outline a framework to measure the success of such a multi-label neural network by introducing molecular F1 score and molecular perfection rate metrics. We hope that others will build-upon our suggested framework and methodology to catalyse further development of functional group identification methods for accurate and autonomous molecular structure elucidation.

## Methods

### Collection of training data

We obtained both FTIR and MS spectra from standard reference spectra published by the United States National Institute for Science and Technology^54^ for 7393 compounds and standardised these spectra using the procedure described in the ESI† under standardisation of FTIR spectra and standardisation of MS spectra.

### Training of neural networks

We used a 3 layered multi-layer perceptron (MLP) network using binary cross entropy as the loss function to allow for multi-label prediction of functional groups. The ReLU activation function was used to introduce non-linearity between layers of the network along with dropout regularisation and batch normalisation to combat overfitting. To train the weights of the model, we applied the Adam optimizer. We applied Five-fold cross validation to ensure a model without overfitting and with minimal bias to training data. All reported validation metrics were averaged over 5 fold and the best hyperparameters were chosen based on these validation metrics. For the autoencoder, a single layer network with an embedding layer of 256 dimensions and ReLU activation was used to encode the spectra. Learned encodings were then given as input to the neural network. The autoencoder helps in removing redundant information and noise from the data. Additional details on training and optimisation of the neural networks presented in this work are mentioned in the ESI† section titled Training and testing of neural networks.

### Assignment of functional groups

We obtained IUPAC InChI strings for all compounds of interest by resolving the CAS number associated with the molecule using the PubChem API.^55^ Then, RDKit^56^ performed substructure matching on each string *via* SMARTS strings to identify the presence of a predefined molecular topology. If a match for a functional group's SMARTS was found, then the compound was deemed a member of the given functional group, and each SMARTS string was tested independently. Therefore, multiple functional groups could be assigned to a single molecule. Initially, we picked functional groups common between those discussed in previous studies.^48,51,52^ These functional groups were chosen to mirror those typically identified using FTIR such that the machine learning model can be analysed to gain insights from learnt chemical patterns, as traditionally done by human chemists. However, it should be noted that more abstract definitions of functional groups can be used in future studies. After training our initial model and analysing the results, we decided to add more functional groups to our model to attempt to improve our results (see Guided backpropagation of the MLP model shows known FTIR and chemical patterns in the Results and discussion for more details). The SMARTS strings used for both models discussed in this work are shown in Table 1 and the distribution of functional groups is given in Fig. S1.†

**Table 1:** SMARTS strings used to identify the presence of a functional group given the 2D topology of a molecule

Functional group	Smarts string
Alkane^a^	[CX4]
Alkene	[$([CX2]=[X2])]
Alkyne	[$([CX2]#C)]
Arene	[c]
Ketone	[#6][CX3](=O)[#6]
Ester	[#6][CX3](=O)[OX2H0][#6]
Amide	[NX3][CX3](=[OX1])[#6]
Carboxylic acid	[CX3](=O)[OX2H1]
Alcohol	[CHX4][OX2H]
Amine	[NX3; H2,H1; !$(NC=O)]
Nitrile	[NX1]#[CX2]
Alkyl halide	[CX4][F,Cl,Br,I]
Acyl halide	[CX3](=[OX1])[F,Cl,Br,I]
Ether^b^	[OD2]([#6])[#6]
Nitro^b^	[$([NX3](=O)=O),$([NX3+](=O)[O–])][!#8]
Methyl^b^	[CH3X4]
Alkane^b^	[CX4; H0,H1,H2]

a The alkane group is redefined in the second set of functional group definitions.

b Groups only present in the second set of functional group definitions.

### Calculation of a molecular F1 metric

**Fig. 2:**
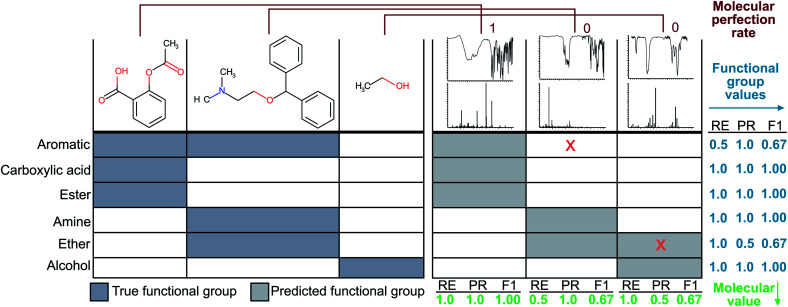
The left-hand side of the figure depicts the ‘true’ functional groups present in the example molecules, and the right-hand side shows example predictions of the molecule functional groups given only their FTIR and MS spectra. Sample calculations for functional group F1 and molecular F1 score are given in the figure. Here, RE is short for ‘recall’ and PR is short for ‘precision’.

Since the correct assignment of all functional groups in a single molecule is paramount to the analysis of organic reactions, we have devised a single metric to quantify the predictive capability of our models *versus* the performance on individual functional groups. Therefore, the focus of our optimisation methodology is to create a model that maximises this overall accuracy measure as opposed to the accuracies of individual functional groups. Similar to the concept of an F1 measure, this metric normalises the performance when the classes (functional groups) are unbalanced. Hence, we have termed this metric the ‘molecular F1 score’ as it describes the success of the model on the whole molecule. This number is calculated for each molecule in the validation set by calculating a ‘molecular precision’ and ‘molecular recall’ value for the functional groups predicted for a given molecule. Precision is the number of functional groups predicted correctly (true positives) divided by the total number of functional groups predicted to be present (the sum of true positives and false positives). Molecular recall is the number of functional groups predicted correctly divided by the total number of actual functional groups present in the molecule (the sum of true positives and false negatives). Similar to the calculation of an F1 score for given functional groups, the molecular F1 is the harmonic mean of the molecular precision and molecular recall. The overall molecular F1 score for a given validation set is the arithmetic mean of all molecular F1 scores. The difference between the molecular F1 and functional group F1 is illustrated in Fig. 2.

### Calculation of a molecular perfection rate metric

While the knowledge of the overall molecular F1 score is useful for comparing models to one another, it does not represent the more stringent criterion of whether a given method produces all functional groups within a given molecule without error. Therefore, we have devised a second metric termed ‘molecular perfection rate’ to rigorously measure the accuracy of our model on a per molecule basis. To calculate this metric, we compare the known functional groups to the predicted functional groups. If the predicted functional groups perfectly match the defined functional groups of the target molecule, then the molecule prediction pair is assigned a molecular perfection of 1; otherwise, it is assigned a molecular perfection of 0. The ‘molecular perfection rate’ for each validation set is calculated as the sum of all individual ‘perfection’ values divided by the total number of molecules. This metric can also represent the percentage of all molecules with a molecular F1 score of 1.0, as shown in Fig. 2.

## Results and discussion

### Multi-layer perceptron neural networks outperform random forest classifiers

We performed an initial computational experiment to determine the choice of machine learning method with the best performance to identify functional groups without carrying out extensive model optimisation. We selected random forest (RF) and multi-layered perceptron (MLP) to test on FTIR spectra to determine if there is a need for using neural networks (MLP) as compared to ensemble methods (RF). An unoptimised MLP consistently outperformed RF models (Fig. S2†) with an average functional group F1-score of 0.771 for the MLP model compared to 0.650 for RF (see Tables S1, S2† for the F1-score of each model and Fig. 2 and the experimental section for definitions of the F1 score). We trained the MLP to predict all functional groups simultaneously as one multi-label classifier. In order to evaluate the effect of transfer learning that has previously been performed for MLP,^43,44,53^ we also evaluated 13 binary classifiers in addition to the 1 multi-label network. The binary classifier approach did not improve the performance of the MLP model significantly as these models only produced an improvement in the functional group F1 score of 0.006 over that of the multi-label model, suggesting that transfer learning is not a significant factor in the multi-label network.

### Multiple functional group prediction in a single compound presents a second optimisation problem

**Fig. 3:**
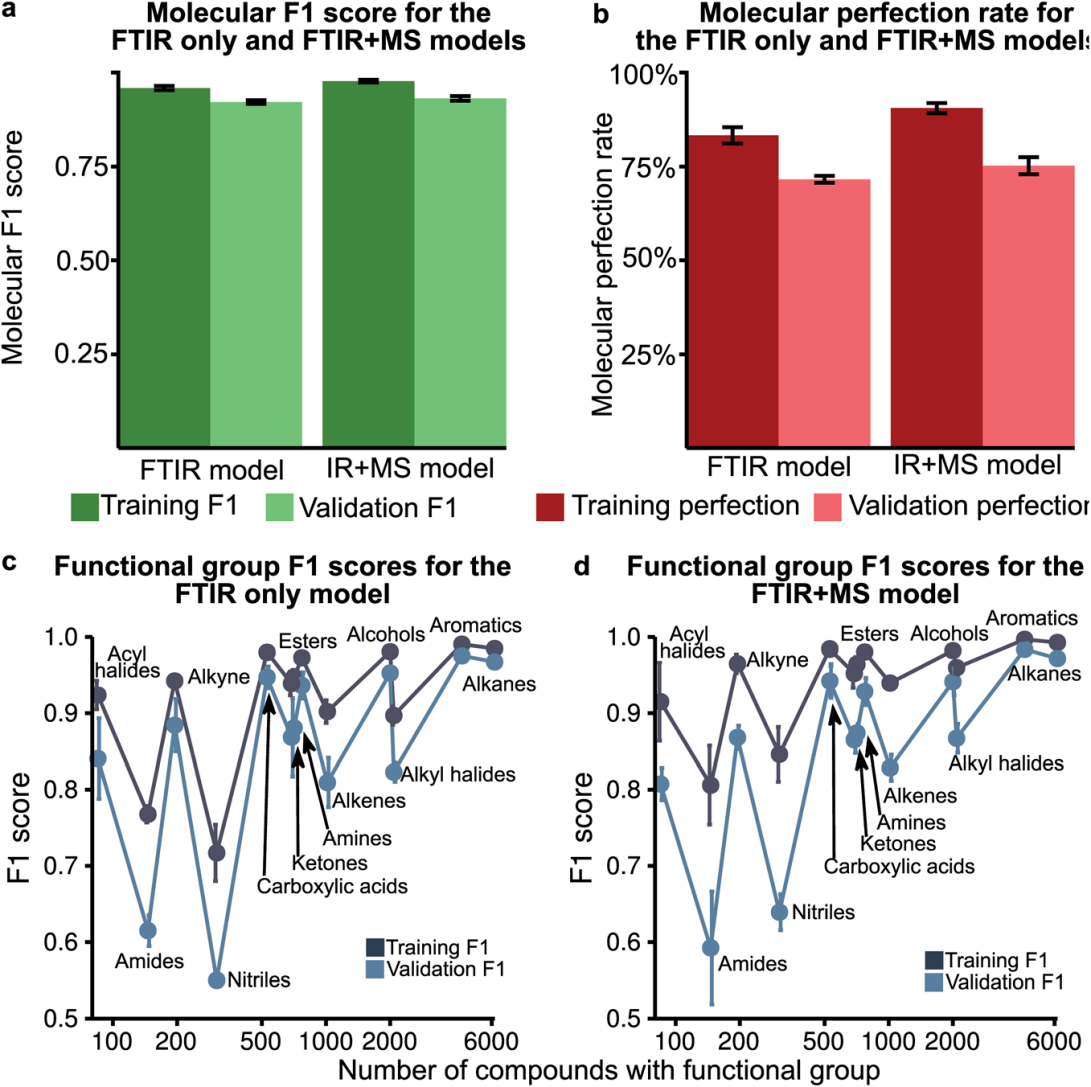
(a) The molecular F1 score for training and validation over 5 fold is shown for both the optimised IR only and IR + MS models. The error bars indicate the standard deviation over the fold. (b) The molecular perfection for training and validation over 5 fold is shown for both the optimised IR only and IR + MS models. (c) The F1 score of the optimised IR only model plotted against the number of occurrences of that functional group. (d) The F1 score of the optimised IR + MS model plotted against the number of occurrences of that functional group.

Analysis of the receiver operator characteristic (ROC) plots (Fig. S3†) shows that at 1% of the false-positive rate, the model identifies over 80% of the true positive functional groups. Therefore, we used a dynamic threshold for each functional group to determine the presence of a functional group in the molecule. This threshold is calculated to maximise the functional group F1 score for the training set after training is complete. While the ability of the model to predict the presence of a particular functional group is important for evaluating the performance of the model, a metric better suited for the study of chemistry and essential for autonomous instrumentation will be to measure the performance of predicting all functional groups in a given molecule. Therefore, we have introduced new metrics, such as the ‘molecular F1 score (MF1)’ and the ‘molecular perfection rate (MPR)’ (see Fig. 2 and the methods section for more details) and optimised our models for the FTIR and FTIR + MS data. After optimisation, the FTIR + MS model was able to perform on par with or better than the optimised combined IR for the majority of functional groups (Fig. 3). The resulting models have comparable average MPRs (72.5% *vs.* 74.9%) and MF1s (0.923 *vs.* 0.931) for FTIR and FTIR + MS, respectively (see Tables S3, S4†). The hyperparameters for these models are given in the ESI† under details of the neural networks.

### MS data addition improves the prediction of specific functional groups

Our optimised MLP model trained on FTIR data performs well on alkanes, ketones, arenes, carboxylic acids, and esters (average validation F1-score of 0.926) but it did not perform at par to predict nitriles, amines, amides, and acyl halides with an average validation F1-score of 0.663 (Fig. 3c, Table S5†). We included the chemical features captured by mass spectrometry (MS) to augment the MLP-FTIR model (Fig. 3d) to address these problematic functional groups. First, we trained an MLP model only on MS data to investigate its predictive capacity for functional groups (Fig. S4a, Table S6†). The difference between the F1 scores of the training set compared to the validation set indicates that MS data need other models for generalisation for consistent performance compared to FTIR data using an MLP architecture (Tables S5, S6†). Similar to the MLP-FTIR model, the MLP-MS model performed well with more data for a given functional group (*e.g.* alkanes, arenes, and alkyl halides) and poorly when fewer data were available (*e.g.* acyl halides, amides, and amines). An additional concern is the low resolution of the MS data with 1 (*m*/*z*) resolution that was used for training the model since this resolution may not be adequate in distinguishing some structures from each other.

Next, we investigated if combining FTIR and MS data could improve *de novo* prediction of functional groups by concatenating spectral data features into an FTIR + MS model (see Experimental section). Table S7† shows the training and validation F1-scores and Table S8† shows the 5-fold result of the MLP model. The improvement of the FTIR + MS model over the FTIR model is presented in Fig. S4b,† and the direct F1 scores are shown in Fig. 3d with an average improvement of 0.024 overall functional groups. However, combining FTIR and MS data results in a substantial increase in validation F1 scores for the nitrile, alkene, and alkyl halide functional groups with improvements of 0.124, 0.048, and 0.061, respectively. The amide functional group remains unchanged as the F1 score is 0.563 for the MLP-FTIR model. The improvement of alkyl halides (Fig. S4b†) may appear to match chemical intuition given the distinct pattern of halogen isotopes observed with MS. However, this conclusion is not supported by the architecture of an MLP model as each input neuron is independent. Future work incorporating the differences in abundance peaks instead of raw values may improve the performance of the MS only model.

### Guided backpropagation of the MLP model shows known FTIR and chemical patterns

**Fig. 4:**
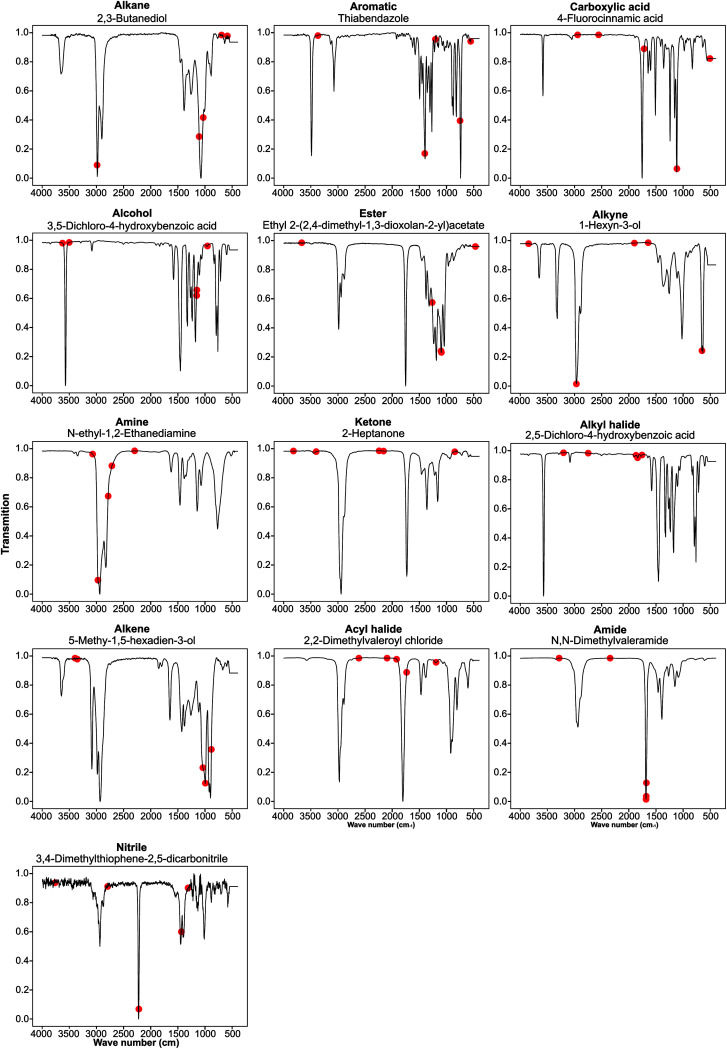
Backpropagation analysis for all 13 functional groups was performed to identify the regions of the spectra responsible for the result obtained. These plots are listed above in order of decreasing F1 score for the optimised FTIR + MS model.

We performed guided backpropagation on the optimised MLP-FTIR model for molecules that were both predicted with an MPR of 1 and have the greatest activation in the neuron corresponding to the respective functional group (Fig. 4). Several backpropagation plots reveal a known chemical association between peaks in FTIR spectra and functional group assignment. This is encouraging as the model was trained without any ‘expert’ or chemical information about the location of the peaks corresponding to each functional group. Specifically, we discuss several functional group cases for our selected set of molecules. The alkane functional group backpropagation shows the use of peaks near 3000 cm^−1^, matching the known location of alkane CH peaks tabulated in the literature. The remaining peaks, however, do not provide any additional chemical intuition with regard to the alkane functional group. Aromatic compounds are identified by a peak between 1400 and 1600 cm^−1^, and the model selected peaks within this region. In addition, the model was able to identify the alkene bending motion around 900 cm^−1^. A C–O stretching is typically observed around 1150 cm^−1^, and the backpropagation plots for carboxylic acids, alcohols, and esters indicate a peak in this region is used by our model for each of these functional groups. Additionally, a strong C=O peak is typically observed for carbonyl compounds near 1600 cm^−1^, but the model only placed importance on this peak for the amide functional group. The example alcohol compound contained both an alcohol group and a carboxylic acid, and the model ignored the C=O in the prediction of the alcohol, instead placing importance on peaks corresponding to the O–H stretching near 3500 cm^−1^. These results show that the model reproduces the ‘known chemistry’ of functional group features without explicit input of peak to functional group relationships.

However, from our chosen set of molecules with an MPR of 1, none of the backpropagation plots revealed any chemically significant characteristics for alkynes, amines, ketones, alkyl halides, and acyl halides. Instead, it appears that these functional groups are identified by the lack of sharp peaks in various regions of the spectra. This observation is interesting as the functional group F1 for these groups is relatively high. While nitrile groups have the lowest performance, the model was able to identify the 2210–2260 cm^−1^ band that is characteristic of this functional group. For the amine functional group, the model places high importance on a peak around 1550–1640 cm^−1^. Although this may appear to indicate learned chemistry since the known N–H bending in this region, it also conflicts with the N–O bending of a nitro group. This observation may explain the reason our model misclassifies many nitro compounds as amides. Fortunately, there is a second N–O bending present which may rectify this issue if we include nitro groups in the model separately.

Next, we investigated the compounds with at least one incorrect functional group prediction (MPR = 0) provided in listing S1.† There are noticeable patterns of functional group types present in the set of failures. One example is nitro groups, which appear over 20 times in the failed compounds. This group is of interest as it is characterised by two strong bands which overlap with bending modes in alkane and amide functional groups. Many of these nitro compounds are misclassified as amides or alkanes and this observation partially explains the poor performance of amide functional groups shown in Fig. 3a and b. Although it is discouraging to note that the model was unable to ‘ignore’ these peaks, the low count of amides present in the dataset may be attributed to this poor performance.

### Additional functional group classification does not affect the model performance of the original definitions

In the previous section, we show that some functional groups explicitly trained in the MLP model were incorrectly classified due to overlapping peaks belonging to functional groups that were not included in our original set of functional group types. We hypothesised that the separate classification of the “overlapping” functional groups could affect the performance of our model. To test this hypothesis, we introduced the ‘nitro,’ ‘ether,’ and ‘aldehyde’ groups into the model. The ‘nitro’ group has significant overlap with the nitrile group (see the previous section), while the ‘ether’ group did not have peak values which overlapped with other functional groups in our previous definition. Another limitation of our model is the inability to distinguish methyl groups from other alkane functional groups. We propose that this is possible due to the lack of a C–C stretching in methyl groups and methyl groups contain characteristic peaks not present in other alkane groups (*i.e.* the CH_3_ bending). In the NIST dataset many alkyl halides are present which do not contain any C–H bonds as all hydrogens in the molecule have been halogenated. Due to the large size of the alkane functional group in the training set, we hypothesise that splitting the alkane group into methyl and ‘other’ alkanes will not result in a large decrease in performance. Therefore, we decided to subdivide the ‘alkane’ group into ‘methyl’ and ‘other’ alkanes as these groups performed the best out of all other groups in the original model.

**Fig. 5:**
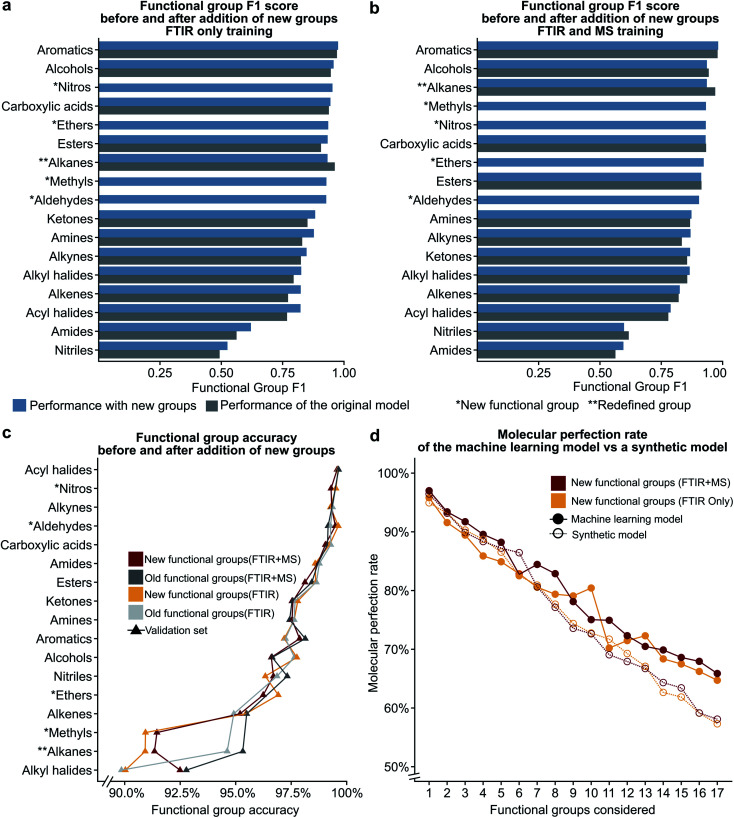
The bar plots given in (a) and (b) compare the functional group F1 scores for the original definitions of functional groups to the new definitions (see Table 1) showing that the addition of new additional functional groups does not have a significant impact on the previous functional groups. The line plot in (c) shows that the accuracy only decreases for the redefined functional group. The plot of molecular perfection rate in (d) compares the performance of the machine learning model to that of a synthetic model to show that the decrease in the molecular perfection rate is expected as the number of functional groups increases.

Fig. 5a–c show the results of these two hypotheses with details presented in Fig. S5 and Tables S9, S10†. The relatively high F1 scores for the ‘methyl’ (0.932) and ‘other’ alkane (0.936) groups support our hypothesis that sub-division of the original alkane definition does not decrease performance. Fig. 5a and b also suggest that our hypothesis to improve low performance of functional groups by the introduction of new functional groups for both the FTIR and FTIR + MS MLP model is incorrect (compare Tables S9, S10 with Tables S3, S5†). Although the nitrile and amide groups do not show improvement after the introduction of the nitro and ether groups as the F1 score for nitriles decreased by 0.019 and amides increased by 0.032, the new groups perform well as compared to the original problematic groups (0.932 for nitro groups and 0.923 for ethers). This suggests that the addition of new functional groups does not cause a significant loss in the F1 score for other groups. Therefore, we speculate that more complex groups could be added to the model to provide detailed structural information, such as a model to identify heterocyclic aromatic rings from rings comprising only carbon. While further subdivision of functional groups is beyond the scope of this work, it presents a potential extension of this work towards realisation of autonomous instrumentation that results in minimal manual intervention.

### Number of functional group predictions affects the molecular perfection rate

We hypothesised that our stringent metric of MPR was affected by the increase in the number of functional group predictions for a given model. To test this hypothesis, we have created synthetic models based on the accuracies of each functional group from the trained FTIR + MS model (see Synthetic models in the ESI† for more details). The machine learning model outperforms these synthetic models (Fig. 5d and S5d†), indicating that increasing the number of functional groups does not decrease this metric more than what would be expected from the inclusion of additional functional groups alone. The overall conclusion of this section is encouraging as it suggests that more functional groups can be added to our model without hurting the model's ability to predict other functional groups. Values for the MPR and MF1 scores for the new functional group definitions are given in Tables S11 and S12.†

**Fig. 6:**
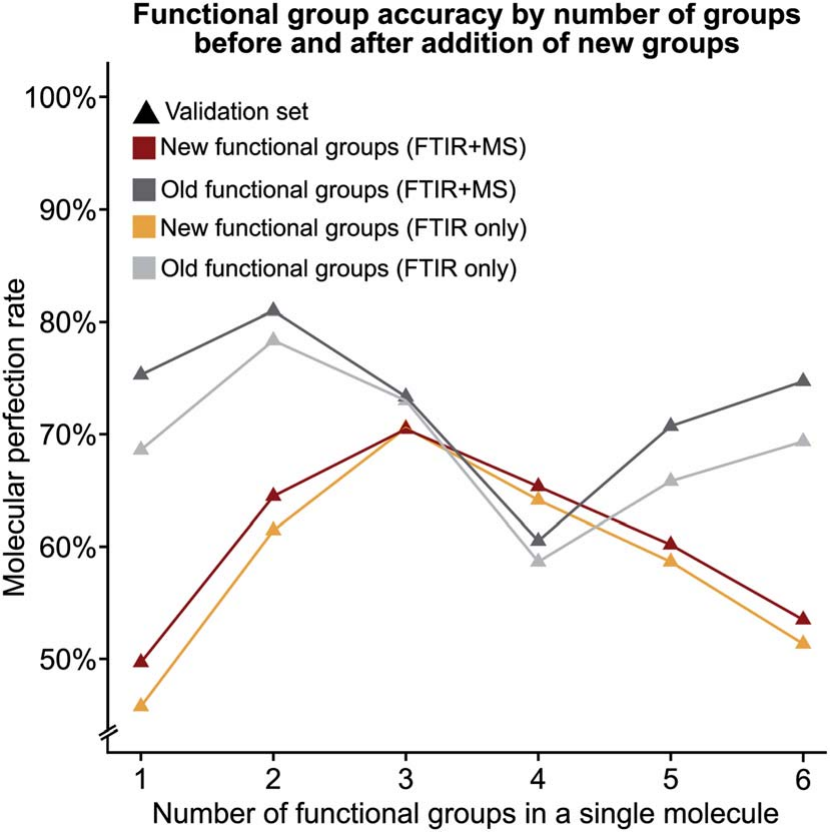
The molecular perfection rate calculated on molecules with a specific number of functional groups for both the original and new set of functional groups.

We were also interested in the performance of our model on molecules with a differing number of functional groups. To do so, we calculated the molecular perfection rate for compounds with one through six functional groups, for the original set of functional groups and the new set of functional groups (results shown in Fig. 6 with details in Fig. S6†). Unfortunately, no definite conclusions can be made from this data as the original *versus* new functional group definitions follow very different patterns. However, the original set of functional groups outperforms the new set of definitions. This observation is likely due to the reduced accuracy of the new alkane due to the split into methyl and non-methyl groups as both have accuracies of 91% where the previous model had an accuracy of 95% (Fig. 5c).

### Encoding spectral data in latent space retains functional group prediction performance

**Fig. 7:**
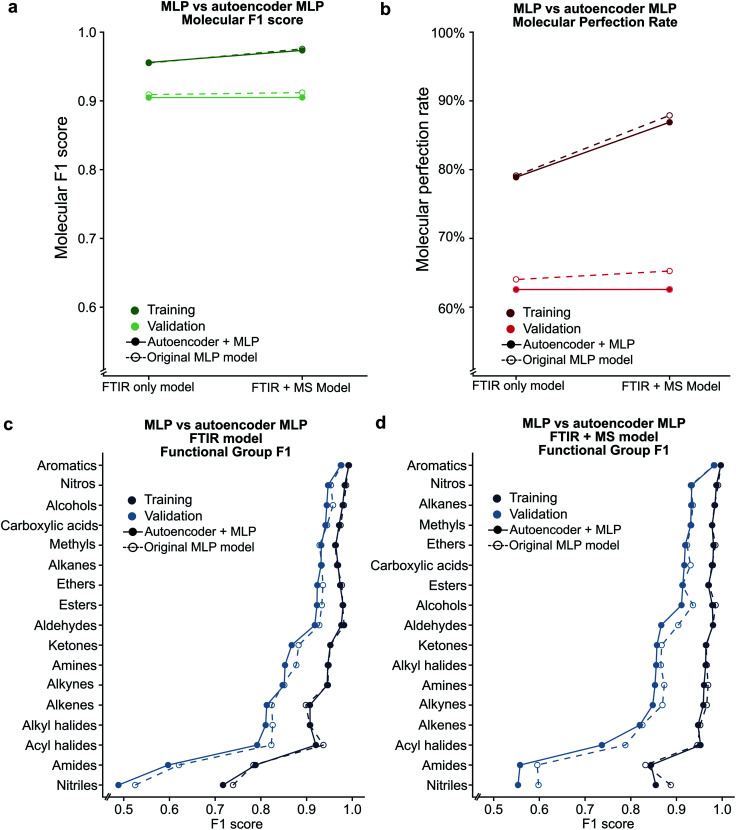
Comparison between the original MLP model and the autoencoder based model using the (a) molecular F1 metric and (b) molecular perfection rate are shown. Individual functional group F1 scores are provided for the FTIR only (c) and FTIR + MS (d) latent spaces.

Given the success of our MLP model in predicting functional groups using complete standardised spectra, we wished to investigate the ability of an autoencoder to reduce the spectra into a latent space. This approach is different from that employed to create the SPLASH keys^57^ for mass spectra. Unlike SPLASH hashed keys, a latent space of spectral data can be uniquely ‘decoded’ back to the original spectra without the use of any external database or additional information. We trained a simple linear model for encoding the FTIR and MS spectra into a 256-length vector and decoding this vector back to the original spectra used to create the vector (see Fig. 1). The 256-length vector was used to train a second network for multi-task functional group prediction. For individual functional groups, the autoencoder model performs similarly to the original MLP model (F1 scores are given in Tables S13 and S14†). The molecular performance of the autoencoder model is similar to that of the original MLP model (Fig. 7) as the MPR for the autoencoder model is 62.6% and the MF1 score is 0.905 as compared to 65.2% and 0.912 for the original model (Tables S15 and S16†). This reveals that the original spectra contain redundant features that relate FTIR and mass spectra. We plan to explore the use of this latent space for inverse design of molecules with combined spectral properties in future work.

### Deep learning model trained on single compounds predicts functional groups in mixtures

The ability to identify all the functional groups in a mixture of compounds expands the applicability of our methodology. To our knowledge, we are the first group to report the ability of machine learning methods to classify mixtures of compounds using a model trained on single compounds. To validate our method on compound mixtures, we obtained the FTIR spectra of three different mixtures of molecules (raw spectra given in Fig. S8–10†) and predicted all the functional groups of the compounds in the mixture using our MLP-FTIR machine learning model (see Table 2). For this test set, we have not included MS data since only a minor improvement was gained from addition of MS spectra based on training. In future work, we plan on improving the performance of functional group prediction by addition of MS data using more advanced machine learning architectures and molecular features. We stress the point that these spectra are obtained in our lab, are not part of the NIST dataset, and are obtained using instruments different from those used by the NIST as it is essential to validate a machine learning method for practical use in different laboratories. Since these spectra are external to the NIST webbook data, they constitute a ‘test set’ for our model. The compound mixtures were prepared by mixing two solid compounds and each mixture contained a different set of functional groups. Performance metrics, such as the molecular F1 score, *etc.*, described previously for single molecules are applied to a mixture of molecules by considering the set of all functional groups (a union of all functional groups present in the mixture). For mixture 1, our FTIR-only method correctly predicted 2 out of the 4 functional groups present in the mixture and predicted an additional functional group not present in the mixture, yielding an MF1 score of 0.65 (Table 2). Given the resolution of spectral data, the lack of an O–H peak above 3500 cm^−1^ could also lead a human chemist to conclude that no carboxylic acid is present in the mixture (Fig. S8†). Additionally, the presence of a peak near 2940 cm^−1^ may lead a human to conclude that a methyl group is present in the mixture (Fig. S8†). For mixture 2, we obtained an MF1 score for the mixture of 0.80 as we correctly predicted 2 out of the three functional groups present in the mixture and did not predict any additional functional groups. The only missed functional group is the amide group, which is known to be problematic in our model (functional group F1 score <0.60) and the lack of a strong peak near 1650 cm^−1^ may contribute to a human's inability to identify this functional group (Fig. S9†). For mixture 3, our method correctly predicted 3 out of the 6 functional groups in the mixture and did not predict any additional groups in the mixture, yielding a molecular F1 score of 0.67. The model was not able to identify a methyl group and a human may make the same mistake given the lack of a peak near 2940 cm^−1^ (Fig. S10†). The model also failed to predict the presence of a nitro group and the presence of an ether, potentially due to the peaks corresponding to these groups overlapping with other peaks in the aromatic region of the spectra. Our results show that the deep learning model trained on single compound spectra can exhibit reasonable performance to predict functional groups for mixtures of compounds. Future work entails training on compound mixture spectral data along with using other deep learning architectures, such as generative adversarial networks. This is essential for correctly estimating the limitations of machine learning models for adoption in industry for autonomous instrumentation.

**Table 2:** Mixtures of molecules used as a test set for the final model

	Mixture 1	Mixture 2	Mixture 3
Component 1	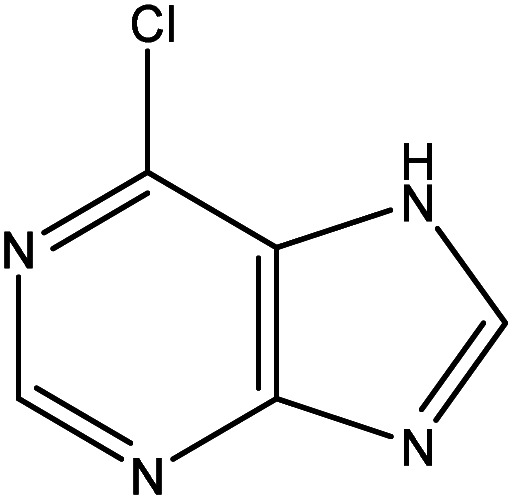	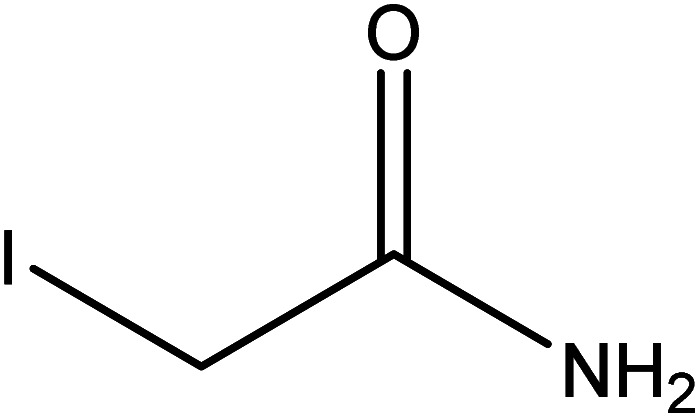	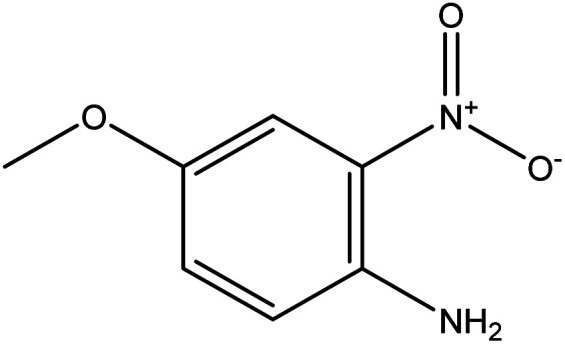
Component 2	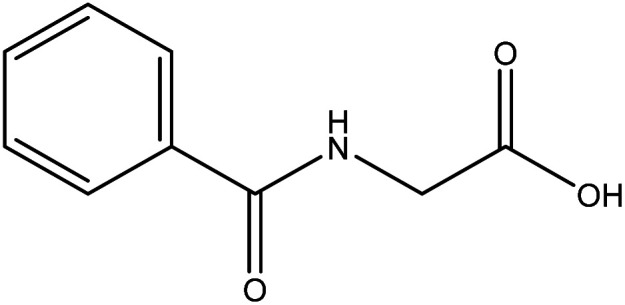	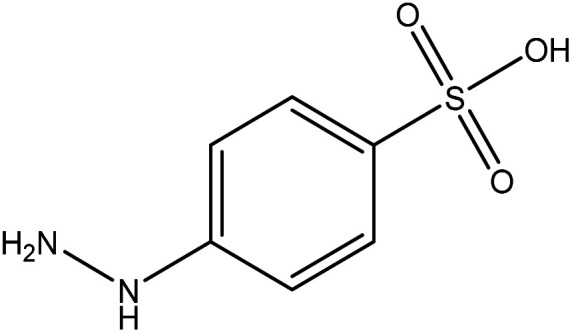	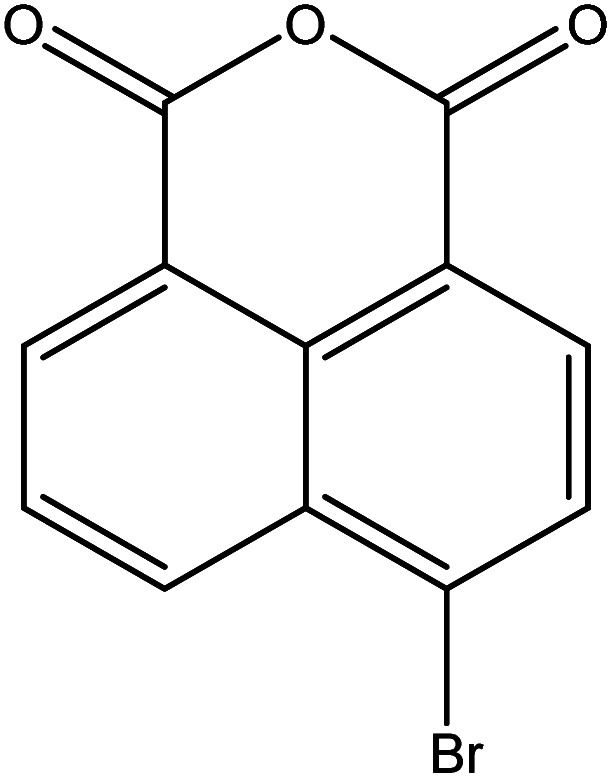
FGs for component 1	Halide, aromatic	Halide, amide	Ether, methyl, nitro, amine
FGs for component 2	Aromatic, amide, carboxylic acid	Aromatic	Halide, aromatic
FGs in mixture	Aromatic, carboxylic acid, halide, amide, alkane	Aromatic, halide, amide	Aromatic, halide, nitro, ether, methyl, amine
Predicted FGs	Aromatic, halide, methyl	Aromatic, halide	Aromatic, halide, amine

## Conclusion

We present a machine learning method for *de novo* prediction of functional groups using a combination of FTIR and MS data. We introduce two new metrics apart from the functional group F1-score, namely molecular F1-score and molecular perfection rate, for practical use of our models. Our results show that, in general, the FTIR data are more consistent for predicting functional groups than MS data, a conclusion backed by chemical intuition. However, several functional group predictions benefit from the inclusion of MS data. Additionally, our model architecture is more optimal for analysis of FTIR data due to the continuous nature of these spectra, and the mathematical structure of an MLP model. Our model's performance is not affected by the number of functional groups present in the training data and it predicted all the functional groups consistently across all metrics. Moreover, several known chemical patterns in the spectra were identified as features for the model to identify common functional groups without any expert training of the system. We conclude that a multi-class, multi-label perspective is apt for further studies which may combine differing spectroscopic data types that may reveal unknown features useful for the identification of compounds. We show that our approach for functional group prediction is flexible as it can be extended to introduce new or sub-divide existing functional groups without affecting performance of original functional group definitions. Furthermore, reducing chemical spectral data in a latent space does affect model performance for predicting functional groups but can be used for inverse design of molecules based on a combination of spectral properties. Finally, we verify that our model also produces reasonable results for a mixture of compounds containing multiple, different functional groups. Therefore, our machine learning model can be used for database-free identification of functional groups in pure and complex mixtures of compounds. We believe that these accomplishments are significant advancements in the development of algorithms and methods for the autonomous identification of functional groups. We hope that the continued development of future spectral learning methods builds upon our work and will adopt or improve upon the molecular F1 score and molecular perfection rate metrics to assess their models to predict multiple functional groups for molecular structure elucidation.

## Data availability

All Python scripts required to build the models used in this work and apply them in the analysis of new spectra are available at https://github.com/chopralab/candiy_spectrum. The NIST Webbook data are copyrighted by NIST and must be obtained from this organisation accordingly.

## Conflicts of interest

The authors declare no conflicts of interest.

## Supplementary Material

SC-011-C9SC06240H-s001
