# Supplementary material for: Spectral deep learning for prediction and prospective validation of functional groups
Source: Chem Sci. 2020 Mar 13;11(18):4618–30. doi: 10.1039/c9sc06240h (PMC8152587; doi:10.1039/c9sc06240h)
Supplement: SC-011-C9SC06240H-s001 [file SC-011-C9SC06240H-s001.pdf]

Electronic Supplementary Material (ESI) for Chemical Science.
This journal is © The Royal Society of Chemistry 2020

Supporting Information

Spectral deep learning for prediction and prospective validation of functional groups

Jonathan A. Fine[1,‡], Anand A. Rajasekar[2,‡], Krupal P. Jethava[1], Gaurav Chopra[1,3,4,5,6,7] *

[1]Department of Chemistry, Purdue University, 720 Clinic Drive, West Lafayette, IN 47906

[2]Indian Institute of Technology Madras

[3]Purdue Institute for Drug Discovery

[4]Purdue Center for Cancer Research

[5]Purdue Institute for Inflammation, Immunology and Infectious Disease

[6]Purdue Institute for Integrative Neuroscience

[7]Integrative Data Science Initiative

[‡]These authors share an equal contribution to this work.

*Corresponding Author

E-mail: gchopra@purdue.edu

Additional Experimental Details

Standardization of FTIR spectra

All FTIR spectra obtained from NIST was truncated so that only peaks occurring from 400cm^{-1} to 4000cm^{-1} remain. The FTIR spectra available in the NIST webbook has varying degrees of resolution, which is problematic for a multilayer perceptron (MLP) network as this architecture requires a discrete and consistent number of input dimensions. To address this, we standardized all FTIR spectra so that each spectrum would have the same number of peaks by defining an FTIR dimension as being the percent transmittance in a wavenumber bin. For example, if a compound has a transmittance of 30% between 400cm^{-1} and 401cm^{-1}, then this dimension has a value of 0.30. Since the most common resolution present in the NIST data is approximately 3.25cm^{-1}, we decided to use this resolution to standardize all FTIR spectra in our dataset. However, other ranges may be better suited for the problem at hand, and the selection of an optimized resolution remains as to be done as future work. To standardize all the spectra, we performed linear interpolation on each IR spectra and evaluated the fitted function at the same set of discrete points throughout each interpolated FTIR spectra. This process yielded uniform FTIR spectra consisting of 1108 points, regardless of the resolution of the original FTIR data.

Standardization of MS spectra

Given the discrete nature of mass spectra, the standardization process for this type of spectra is straightforward. The bin size for these spectra was chosen to be 1 m/z unit, and the counts present in each bin were averaged together for spectra with a resolution less than 1 m/z. All the bin counts in each spectrum were divided by the largest count in the same spectrum to yield the relative abundance for all the m/z peaks present in the dataset. This resolution was selected as it is the best resolution provided for all spectra available in the NIST webbook.

Training and testing of neural networks

All Neural Networks reported in this work were created using the Keras Python Package[7]. All hidden layers were normalized using batch normalization and activated using rectified linear units, and a sigmoidal function is used to activate the final output layer. During the training procedure, we applied a dropout procedure which randomly selects neurons given a per layer probability and sets their value to zero. We used binary cross entropy was used as the loss function for training the neural network as this loss function is standard for multi-label classification problems. For each epoch of training, Keras calculated the loss of the training and validation sets and compared this loss to the loss of the previous epoch. Early stopping with a patience of 5 epochs was used to prevent the model from overtraining. All models were validated using 5-fold cross-validation, and sequential hyperparameter searching was used to optimize the final FTIR and MS model. The hyperparameters of the optimized model are given in the supporting information under Details of neural networks.

The overall mathematical representation of this model can be represented with Formula 1 given below.

$$\vec{y} = f\left(\vec{a}^{0}, W, \vec{b}\right) \tag{1}$$

Here, , $\vec{y}$ is the predicted functional groups from the model with a length equal to the number of functional groups defined in the previous section. Each component of this vector represents the probability that the corresponding functional group is present in the molecule. The vector $\vec{a^0}$ is the input spectra and has a length equal to the number of components in the spectra. Matrix W is a weighting matrix and $\vec{b}$ is bias a vector. All terms are applied in the following manner:

$$f\left(\vec{a^0},W,\vec{b}\,\right) = \sigma\left(W\vec{a^0}\ + \vec{b}\right) \tag{2}$$

This function can be applied. multiple times with matrices of varying length, producing hidden 'layers.' The optimal number of layers, as well as their respective lengths, are 'hyperparameters.' For each neuron k in a layer l:

$$a_j^l = \sigma\left(\sum_k w_{jk}^l a^{l-1}_k + b_j^l\right) = \sigma\left(z_j^l\right) \tag{3}$$

Here, σ is an activation function. For hidden layers:

$$\sigma = ReLU(x) = max(0,x) \tag{4}$$

For the final layer:

$$\sigma = sigmoid(x) \tag{5}$$

The cost function, or error in the model, is defined as follows using the binary cross entropy function:

$$C(\vec{y},\tilde{y}) = \tilde{y}\log\vec{y} + (1 - \tilde{y})\tilde{y}\log\left(1 - \vec{y}\right) \tag{6}$$

Where $\vec{y}$ is the predicted functional groups and $\vec{y}$ are the true functional groups. The goal of back propagation is to minimize the cost function, thereby making the model increasingly accurate.

We define the error of any neuron to be

$$\delta_j^l = \frac{\delta C}{\delta z_j^l} \tag{7}$$

For the final layer of the model, we can compute this value via the chain rule:

$$\delta_j^L = \frac{\delta C}{\delta a_j^L}\sigma'(z_j^L) \tag{8}$$

This expression can be rewritten as the following for a matrix operation:

$$\delta^L = \nabla_a C \odot \sigma'(z^L) \tag{9}$$

Once the derivative of the final layer is obtained, the derivative of the penultimate layer can be calculated as follows:

$$\delta^l = \left(\left(W^{l+1}\right)^T \delta^{l+1}\right) \odot \sigma'(z^l) \tag{10}$$

One can continue to 'backpropagate' this derivative until the derivative of the first layer is calculated. Now that δ_j^l can be calculated for all layers, the derivate of the total cost function with respect to a bias term can be written as

$$\frac{\delta C}{\delta b_j^l} = \delta_j^l \tag{11}$$

Additionally, the following for the weighting terms:

$$\frac{\delta C}{\delta W_{jk}^{l}} = a^{l-1}_{k}\delta_j^l \tag{12}$$

Now that all backpropagation terms can be calculated for all layers, we can define a modified version of this procedure referred to as guided backpropagation. This procedure begins with an already-trained network consisting of W and $\vec{b}$. The network is predicted in the forward direction to obtain $\vec{y}$. Then equations (9) and (10) are used to calculate the weights of the input vector $\vec{a}^0$. Note that all negative gradients are set to 0 during the application of (10). Details for the application of guided backpropagation in this work are given in the following section.

Use of backpropagation to identify patterns in the model

One of the most challenging problems of developing deep learning models is interpreting them in a chemical context[8], which results in many researchers treating these models as black-box representations, thereby neglecting to understand how features are used to predict results. Solving this problem to understand how various input features result in a given result is a challenging problem. Many new methods were proposed to address this problem which can be broadly classified into perturbation-based methods and backpropagation-based methods. Back propagation-based methods compute the gradient of the activations concerning the feature space and identify the section of the image which maximally activates that neuron. One such backpropagation based method is 'guided backpropagation[9].' Guided backpropagation is similar to the 'probability' approach[10], where the difference between these two methods arises from differences in the handling of backpropagation. The deconvnet approach computes the gradient-based on top gradient signal setting negative values to zero while backpropagation sets the gradient of negative activations in the forward pass to zero; avoiding the 'flow' of negative gradients during backpropagation. We use this technique to identify the top 5 bins of an input FTIR spectra that was responsible for predicting a particular functional group of a molecule and present these results in Figure 4.

General Information:

FTIR spectra were recorded by ATR-IR method using Thermo Nicolet FTIR instrument and processed using OMNIC software. The compound mixtures were prepared by mixing two solid compounds (crushed with spatula to make fine powder, if necessary) and used directly for ATR-IR. MS spectra were recorded by Electron Spray Ionization method (ESI) using Agilent 6470 triple quadruple LC/MS. Solvents and commercially available compounds were used as it is without any further purification.

Synthetic Models

We developed a control, which we have termed as 'synthetic models', for this work which are created using a predefined accuracy to assign functional groups. To generate a synthetic model, one takes the original functional group matrix (where columns are functional groups and rows are molecules) and predicts each functional group for every molecule individually based on random numbers. The accuracy of each synthetic model is fixed, and the predictions are randomly assigned as correct or incorrect to obtain the specified accuracy. Unlike a truly random model, the synthetic model has access to the original functional group assignment matrix and the predictions of the matrix are not randomly assigned but are instead 'purposefully' correct or incorrect based on a uniform random distribution. For example, consider a synthetic model that has an accuracy of 50% and is being generated for 4 functional groups. It is given a molecule where only the first 2 functional groups are present ([1,1,0,0]). Four random numbers are generated using a uniform distribution, e.g.: 0.25, 0.75, 0.85, and 0.10. Since the second and third random numbers are greater than the assigned accuracy (0.50), they are deemed incorrect and the model will predict ([1,0,1,0]). This example has a molecular recall of 0.5, a molecular precision of 0.5 and molecular perfection of 0.

Synthetic models with accuracies of 99%, 95%, and 90% are given below showing decrease in MPR with increase in number of functional group predictions.

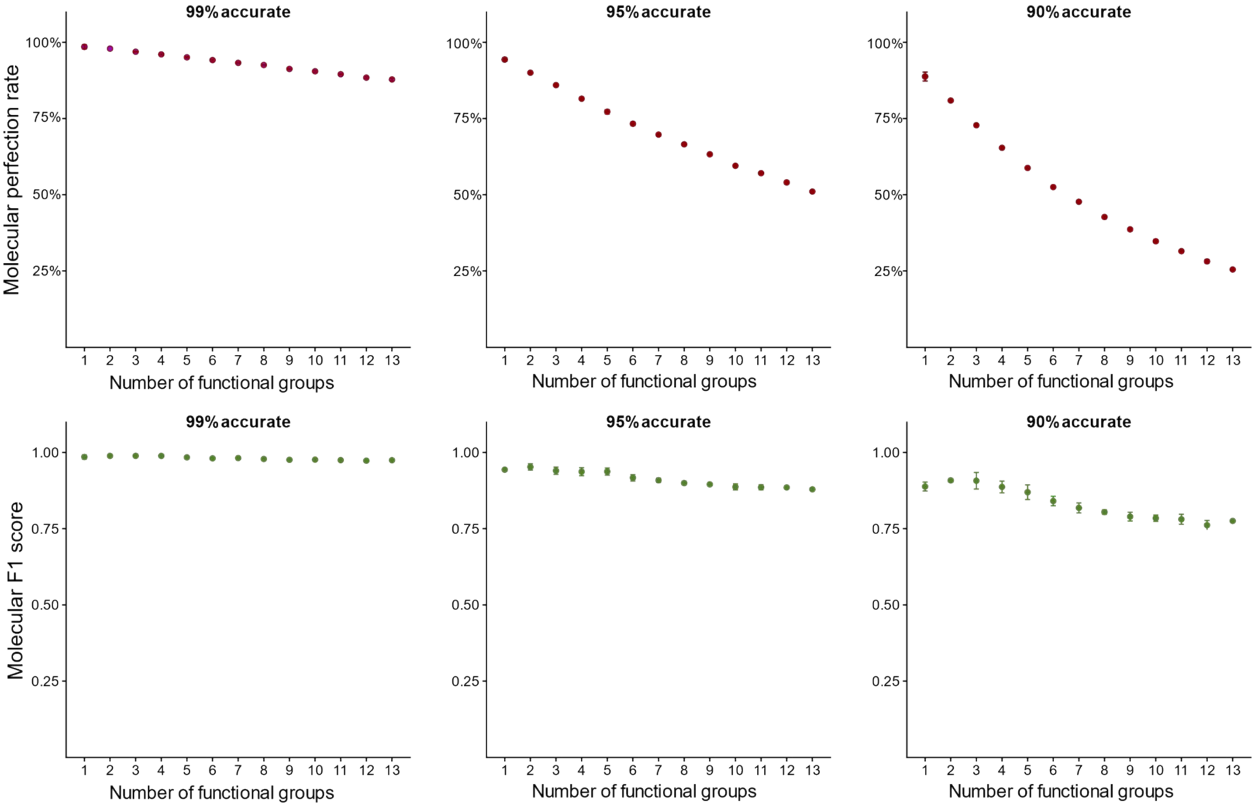

The synthetic models shown in **Figure 5d** and **Figure S5d** are created to evaluate how molecular perfection rate varies with the introduction of additional functional groups to a machine learning model. Individual synthetic models with differing numbers of functional groups (X-axis) are created, and the corresponding molecular perfection rates are calculated for each synthetic model. Since the purpose of a synthetic model is to serve as a comparison to a machine learning model, the accuracies for each synthetic model is the average of a subset of functional group accuracies taken from the comparison model. These functional group subsets are selected at random every time a synthetic model is created. For example, consider a machine learning model with functional group accuracies of 90%, 80%, and 70%; this allows for three synthetic models with two functional groups to be created with accuracies of 85%, 80%, and 75% and a single synthetic model with three functional groups to be created with an accuracy of 80%. For all figures produced in this work, 10 synthetic models are created for each number of functional groups considered (ticks on the X-axis), and the average molecular perfection rate of these 10 models are plotted on the Y-axis.

Details of neural networks

The final optimization parameters for the FTIR+MS model:

Layer size	**Dropout**
237	0.457866692938781
170	0.26437107014663824

Batch size: 178.0

For the FTIR model:

Layer size	**Dropout**
240	0.3820803111613069
200	0.38822353533309584
131	0.008815281710900874

Batch size: 153.0

Supporting Figures

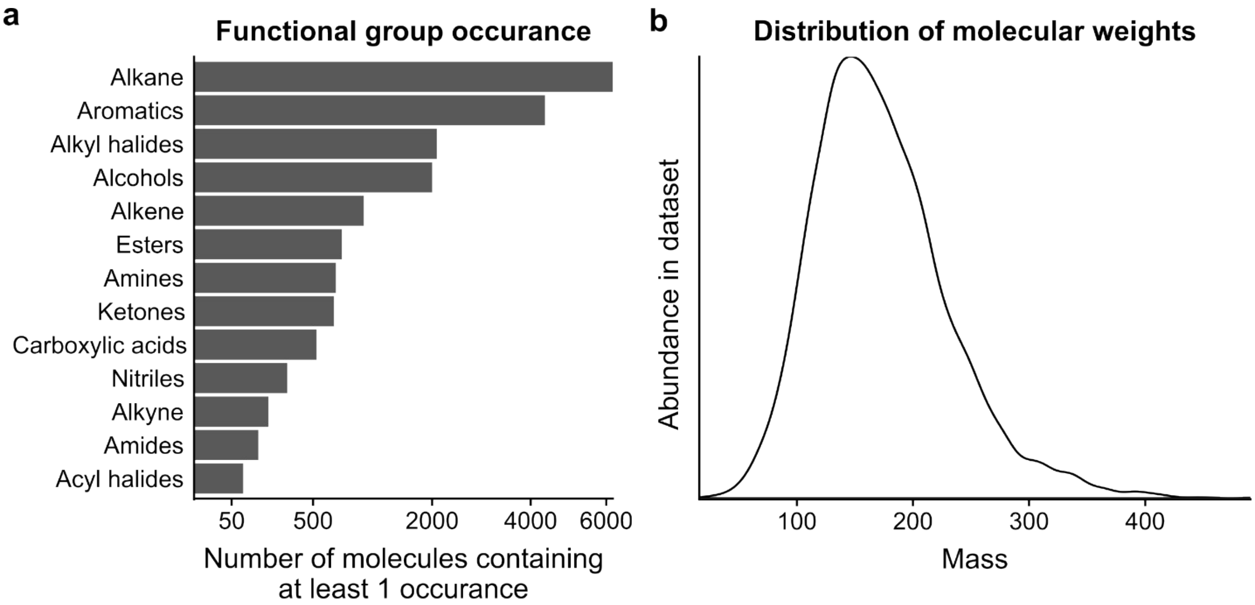

Figure S1: (a) The distribution of various functional groups in the NIST database. (b) The distribution of molecular masses present in the NIST database.

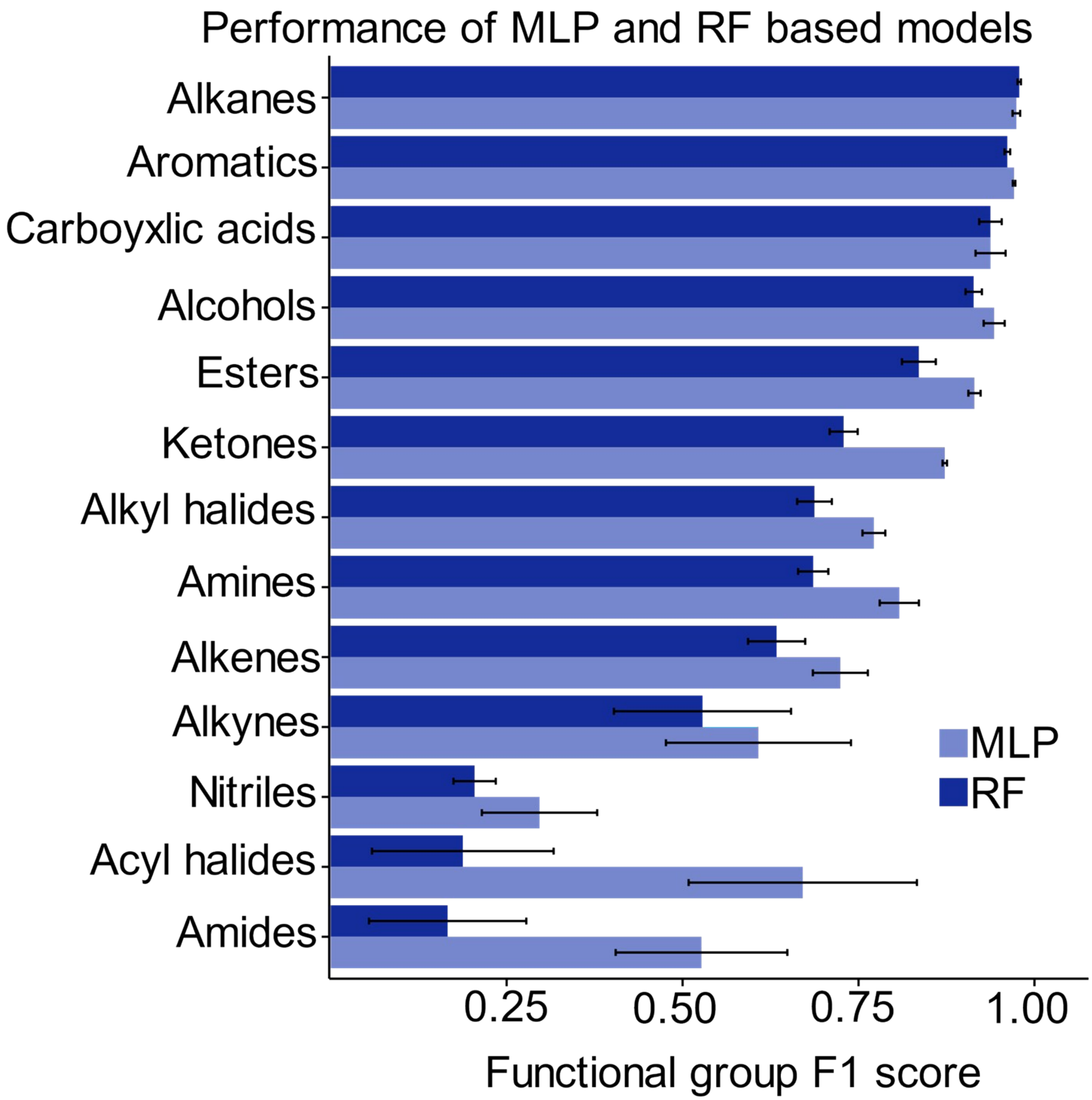

Figure S2: The comparison of Random Forest and Multi-Layered Perception validation set performance for the selected functional groups indicates that the MLP methodology outperforms RF for the majority of functional groups. Both methods were trained on the FTIR spectra only and no hyperparameters were used to optimize the model. Each bar represents the mean of a 5-fold cross-validation, and the error bars indicate the standard deviation over the 5-folds. Here, the MLP model outperforms random forest and this is apparent for amides, acyl halides, amines, alkyl halides, ketones, and esters.

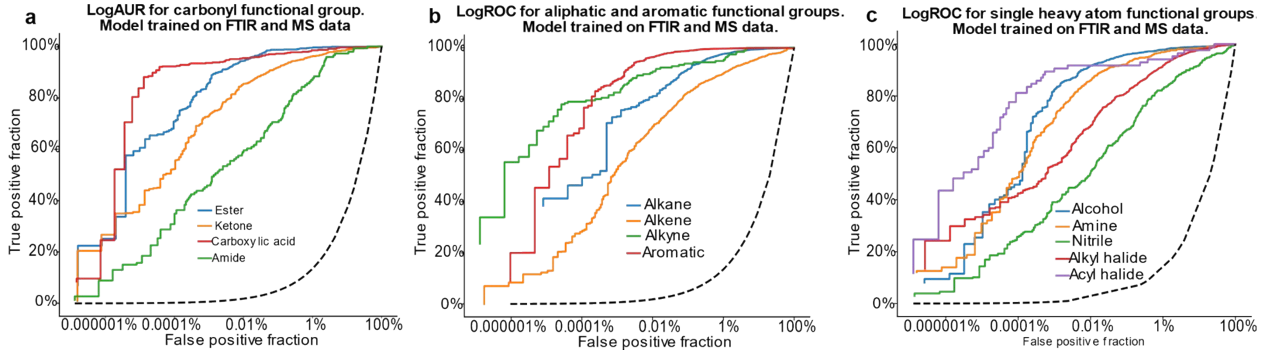

Figure S3: Receiver Operator Characteristic (ROC) plots for the model trained on both FTIR and MS spectra. (a) performance for carbonyl functional groups, (b) groups consisting of only carbon and hydrogen, and (c)the remaining functional groups. The underperformance of amides and nitriles can be discerned from these plots. These plots also allow us to select the best threshold value for each functional group which maximizes the F1 score for that functional group.

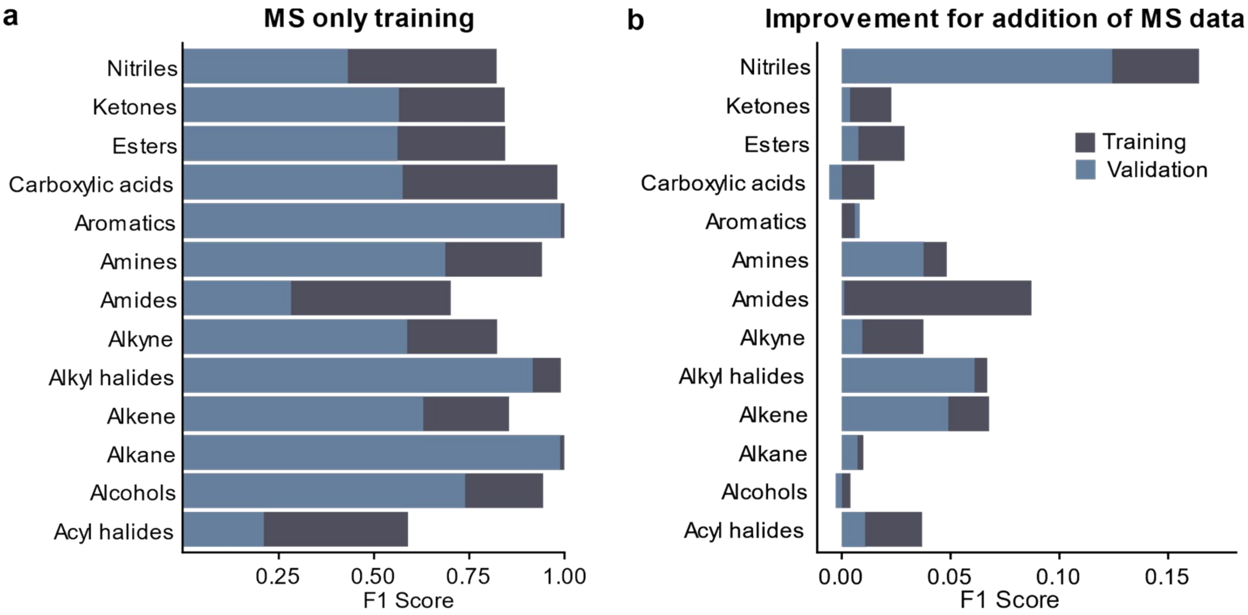

Figure S4: (a) Per functional group performance for an MLP model trained only on MS data shows that the model trained only FTIR data outperforms the model trained only on MS data during K-Fold validation. Also, the MS only model tends to become overtrained in comparison to the FTIR model potentially due to a greater degree of generalization for FTIR data. (b) The improvement in performance for each functional group when MS spectra are introduced in addition to FTIR data.

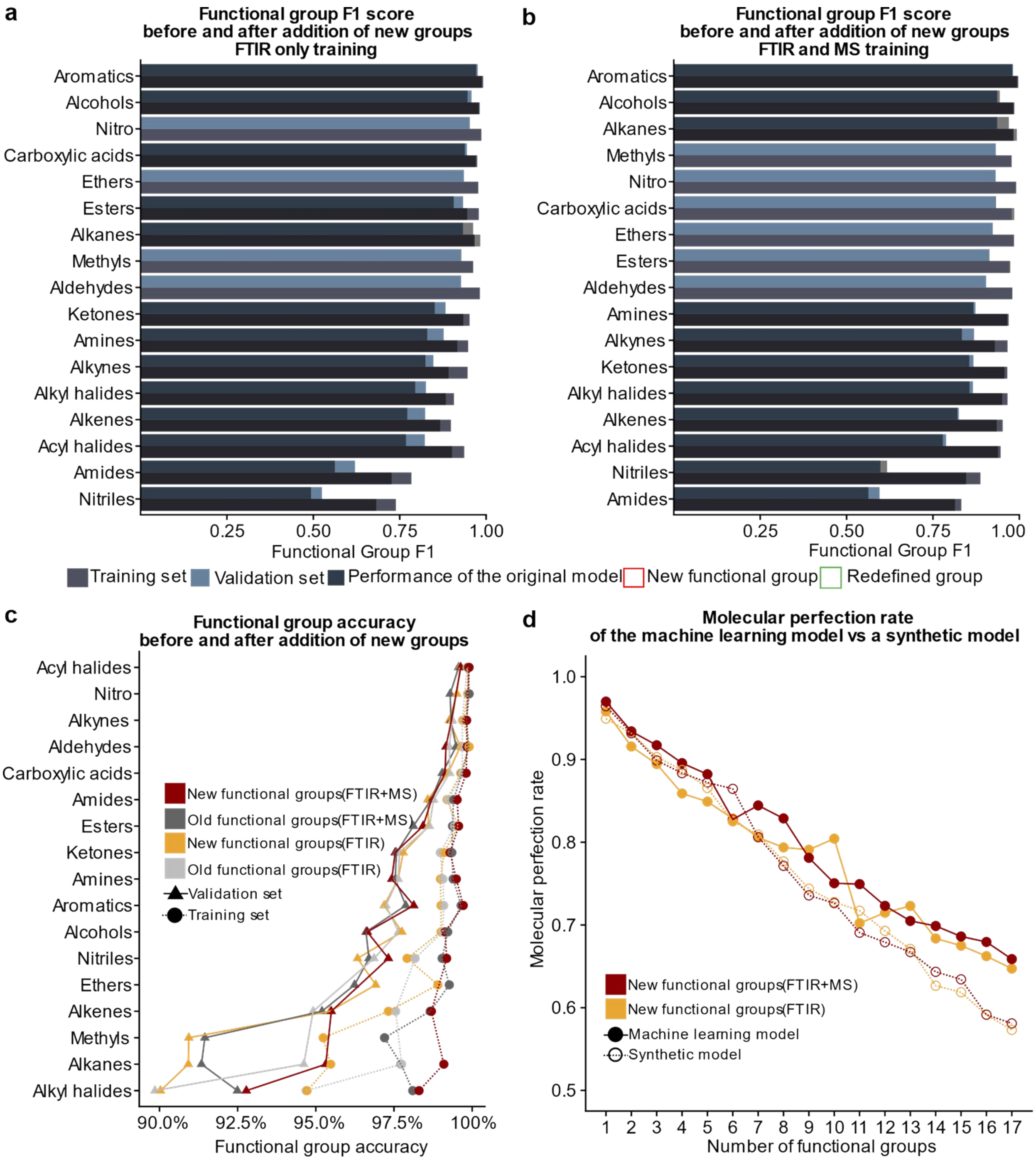

Figure S5: A extended version of **Figure 5** in the main text. Here, we show both training and validation set results for panels **a-c** in the original figure.

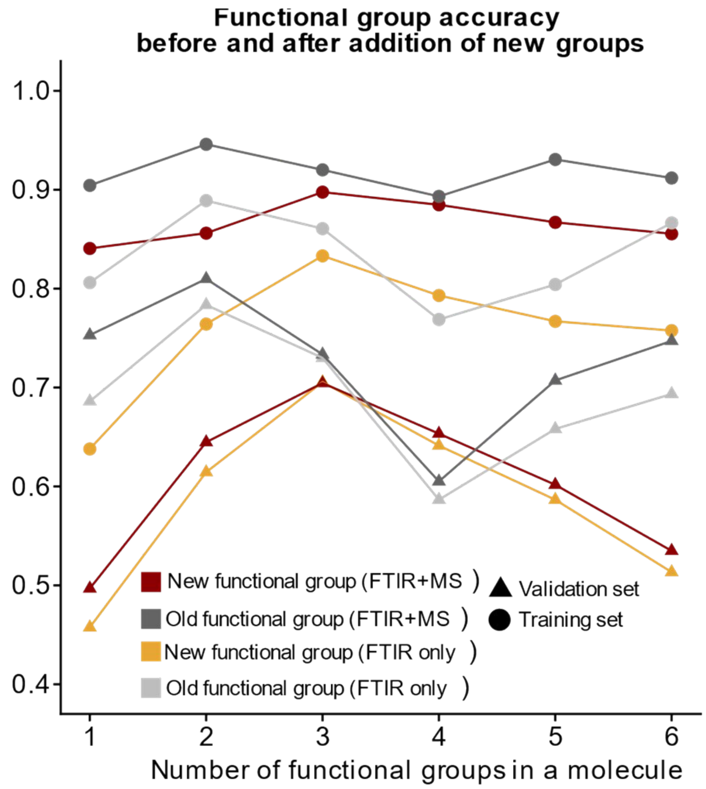

Figure S6 The molecular perfection rate calculated on molecules with a specific number of functional groups for both the original and new set of functional groups.

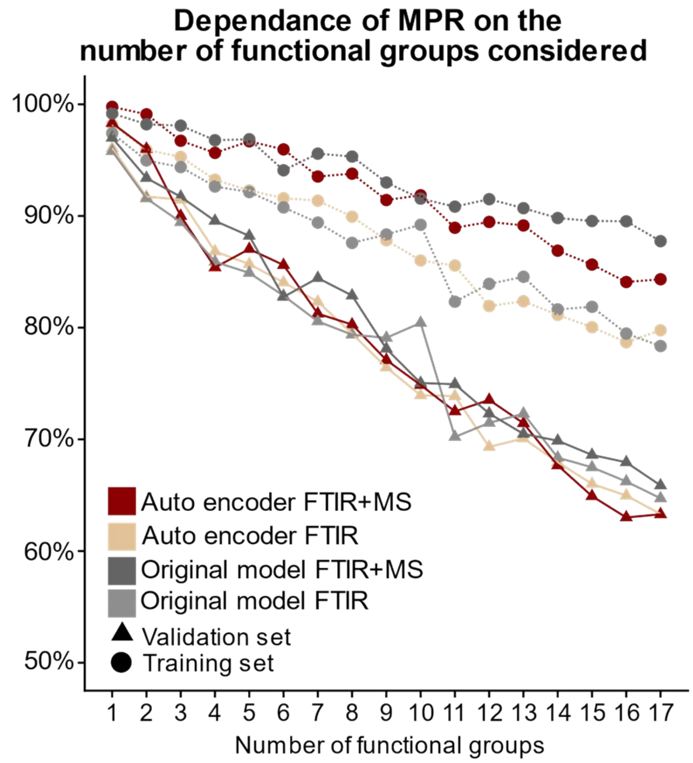

Figure S7: A version of **Figure 5d** which includes both the training set data and the autoencoder model. This figure shows that the autoencoder and training set data follow the same trend as the one found in the original model's validation set.

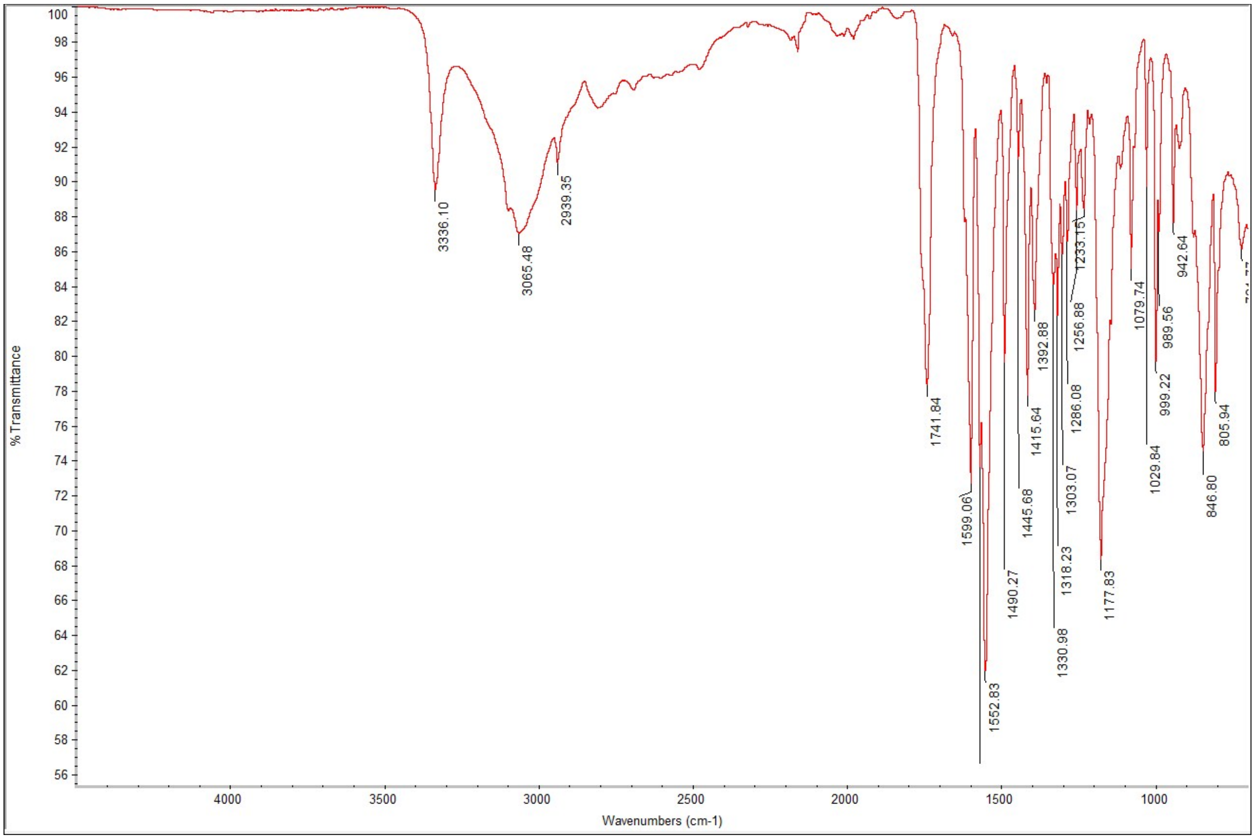

Figure S8: Recorded ATR-IR spectrum for mixture 1.

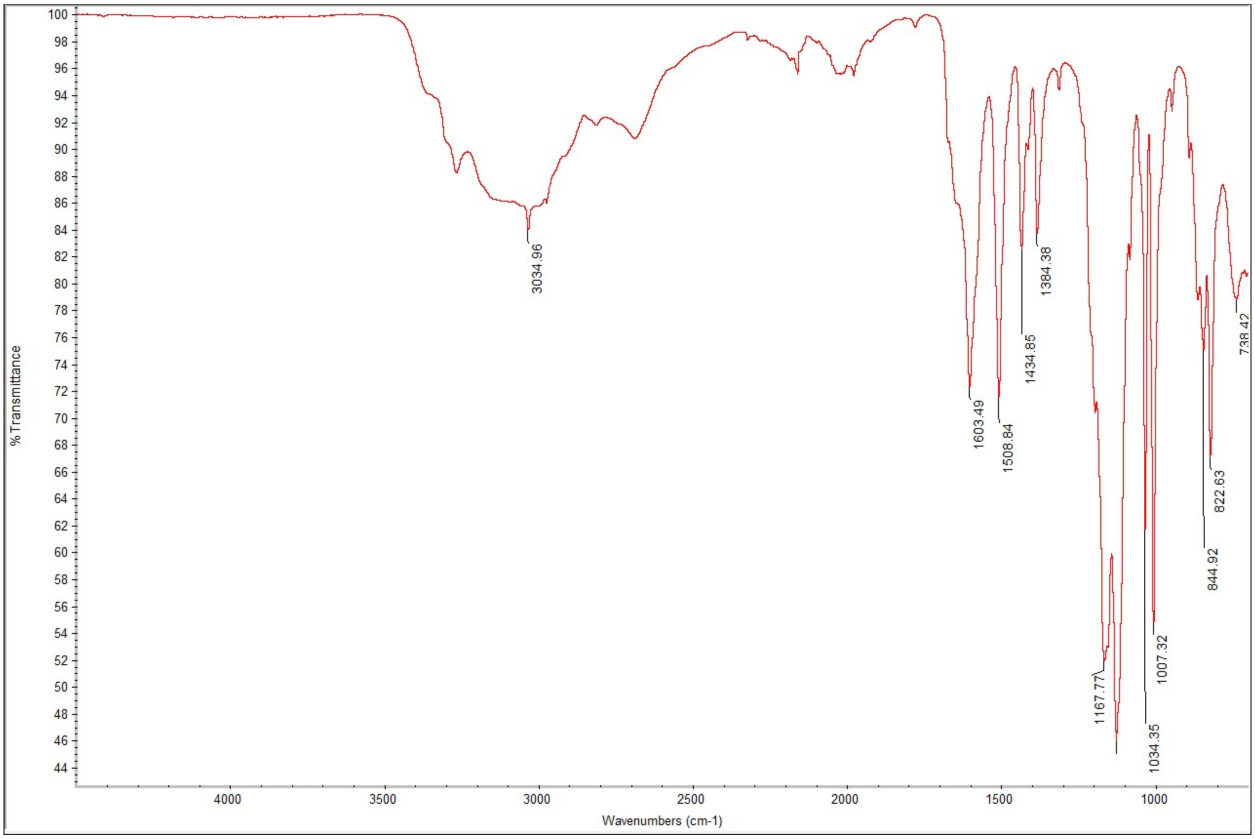

Figure S9: Recorded ATR-IR spectrum for mixture 2.

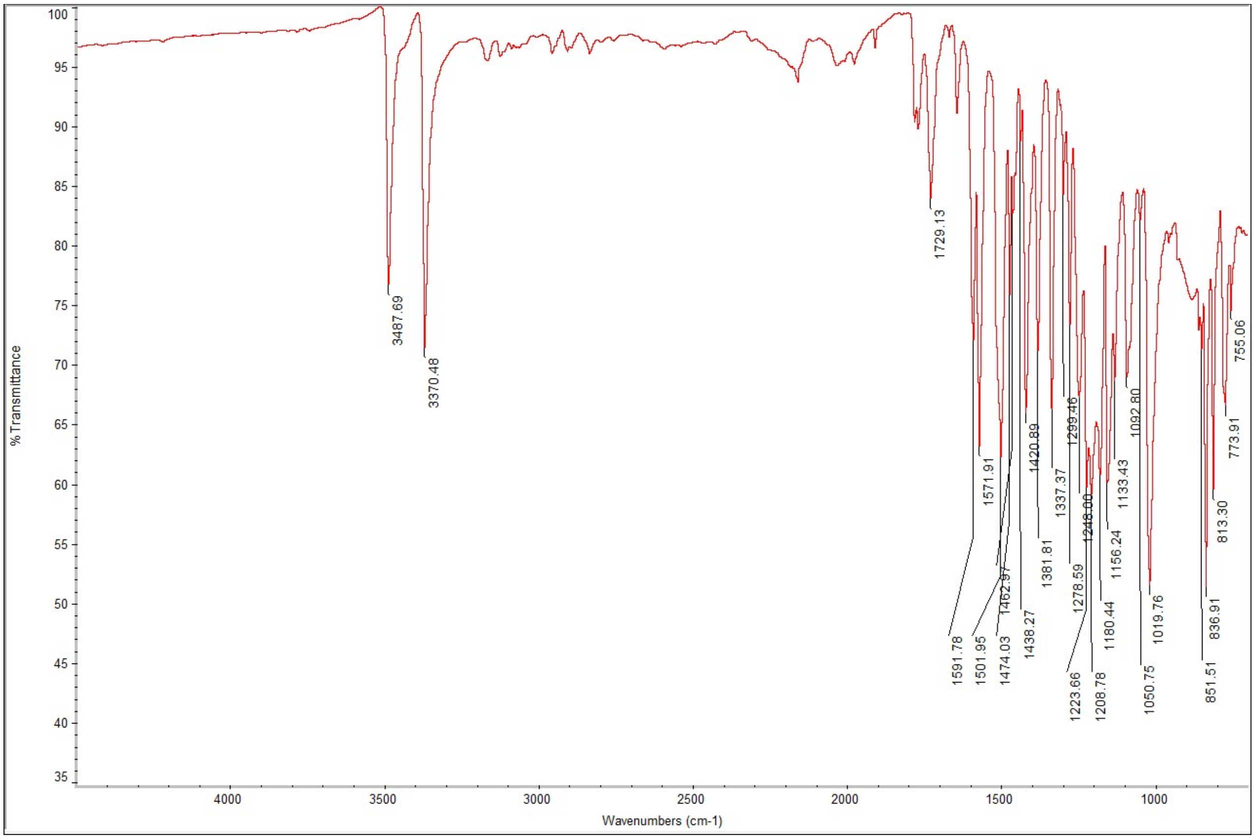

Figure S10: Recorded ATR-IR spectrum for mixture 3.

Supporting Tables

Functional Group	Fold1	Fold2	Fold3	Fold4	Fold5
Alkane	0.979032	0.976801	0.975889	0.978278	0.982206
Alkene	0.648829	0.616949	0.578778	0.634441	0.689139
Alkyne	0.367347	0.629630	0.509804	0.458333	0.676471
Alcohols	0.902405	0.900000	0.919127	0.922293	0.924731
Amines	0.647303	0.695652	0.692982	0.695652	0.696517
Nitriles	0.246154	0.222222	0.181818	0.200000	0.172414
Aromatics	0.957637	0.958546	0.959868	0.965123	0.966496
Alkyl halides	0.679083	0.701001	0.723837	0.665765	0.667638
Esters	0.805556	0.842105	0.860215	0.816000	0.855072
Ketones	0.757991	0.720000	0.736364	0.725664	0.704663
Carboxylic acids	0.931217	0.944724	0.928230	0.921569	0.961905
Acyl halides	0.222222	0.105263	0.100000	0.400000	0.111111
Amides	0.050000	0.258065	0.071429	0.303003	0.148148

Table S1: Functional group F1 scores for the random forest model.

Functional Group	Fold1	Fold2	Fold3	Fold4	Fold5
Alkanes	0.969475	0.979757	0.970094	0.971729	0.980640
Alkenes	0.689459	0.728291	0.706849	0.706849	0.789644
Alkynes	0.423077	0.678571	0.677966	0.520000	0.739726
Alcohols	0.923497	0.932015	0.955446	0.944649	0.958333
Amines	0.788530	0.776371	0.847826	0.807407	0.819672
Nitriles	0.238806	0.243243	0.289157	0.273973	0.438356
Aromatics	0.974011	0.969907	0.969461	0.971098	0.970862
Alkyl halides	0.768229	0.749672	0.786842	0.788698	0.765743
Esters	0.926380	0.916944	0.903427	0.909747	0.917492
Ketones	0.876404	0.874172	0.868526	0.873563	0.870293
Carboxylic acids	0.953846	0.920000	0.939535	0.912621	0.962617
Acyl halides	0.416667	0.714286	0.689655	0.666667	0.866667
Amides	0.333333	0.600000	0.550000	0.651163	0.500000

Table S2: Functional group F1 scores for the initial neural network model.

	Molecular Perfection Rate	Molecular F-1
**Training set**	85.2767%	0.963177
**Validation set**	72.5011%	0.923357

Table S3: MPR and MF1 values for a multitask model trained on only FTIR spectra

	Molecular Perfection Rate	Molecular F-1
**Training set**	92.5571%	0.982041
**Validation set**	74.9085%	0.931506

Table S4: MPR and MF1 values for a multitask model trained on FTIR and MS spectra

Functional Group	Training set F1	Validation set F1
**Alkane**	0.983057	0.962597
**Alkene**	0.866956	0.771962
**Alkyne**	0.891495	0.824410
**Alcohols**	0.978567	0.946291
**Amines**	0.916645	0.829724
**Nitriles**	0.682049	0.493131
**Aromatics**	0.987723	0.971455
**Alkyl halides**	0.883381	0.794842
**Esters**	0.945287	0.906326
**Ketones**	0.933960	0.851401
**Carboxylic acids**	0.970379	0.938528
**Acyl halides**	0.901172	0.767982
**Amides**	0.726499	0.562125

Table S5: Functional group F1 scores for the neural network model trained on only FTIR spectra

Functional Group	Training set F1	Validation set F1
**Alkane**	0.999203	0.988438
**Alkene**	0.854542	0.629076
**Alkyne**	0.823007	0.586774
**Alcohols**	0.944003	0.738918
**Amines**	0.940923	0.686877
**Nitriles**	0.821942	0.431075
**Aromatics**	0.999599	0.990165
**Alkyl halides**	0.990067	0.916458
**Esters**	0.844197	0.561388
**Ketones**	0.843226	0.565298
**Carboxylic acids**	0.981144	0.574958
**Acyl halides**	0.589059	0.210796
**Amides**	0.701243	0.282036

Table S6: Functional group F1 scores for the neural network model trained on only MS spectra

Functional Group	Training set F1	Validation set F1
**Alkane**	0.992912	0.969812
**Alkene**	0.934595	0.820889
**Alkyne**	0.928958	0.833759
**Alcohols**	0.982569	0.943450
**Amines**	0.964915	0.867276
**Nitriles**	0.846275	0.617405
**Aromatics**	0.993621	0.979649
**Alkyl halides**	0.950192	0.855821
**Esters**	0.974097	0.913863
**Ketones**	0.956765	0.855171
**Carboxylic acids**	0.985346	0.932786
**Acyl halides**	0.938061	0.778668
**Amides**	0.813684	0.563190

Table S7: Functional group F1 scores for the neural network model trained on both FTIR and MS spectra

Functional Group	Fold1	Fold2	Fold3	Fold4	Fold5
**Alkane**	0.975453	0.97485	0.977956	0.984628	0.971593
**Alkene**	0.820779	0.796657	0.813648	0.782396	0.833333
**Alkyne**	0.818182	0.852941	0.857143	0.900000	0.707071
**Alcohols**	0.951654	0.931789	0.932515	0.931436	0.929397
**Amines**	0.879433	0.879433	0.879699	0.853047	0.861314
**Nitriles**	0.647059	0.715596	0.533333	0.637931	0.632653
**Aromatics**	0.979167	0.982739	0.978748	0.984438	0.982416
**Alkyl halides**	0.871338	0.849449	0.878107	0.881748	0.875931
**Esters**	0.929487	0.900000	0.914286	0.924528	0.923588
**Ketones**	0.853755	0.892308	0.817814	0.881890	0.828571
**Carboxylic acids**	0.92093	0.900990	0.955665	0.940594	0.897561
**Acyl halides**	0.785714	0.857143	0.833333	0.838710	0.709677
**Amides**	0.612245	0.640000	0.478261	0.612245	0.690909

Table S8: Functional group F-1 scores for single neural networks trained on both FTIR and MS spectra

Functional Group	Training set F1	Validation set F1
Alkanes	0.966563	0.932969
Alkenes	0.898341	0.823709
Alkynes	0.946598	0.847545
Alcohols	0.981538	0.957765
Amines	0.948083	0.877436
Aitriles	0.739183	0.525128
Aromatics	0.991503	0.976025
Alkyl halides	0.907264	0.825761
Esters	0.978914	0.933366
Ketones	0.952114	0.882585
Aldehydes	0.982074	0.927797
Carboxylic acids	0.974353	0.944752
Acyl halides	0.936764	0.822867
Amides	0.783791	0.620740
Methyl	0.962598	0.928545
Ether	0.977310	0.935875
Nitro	0.986419	0.953173

Table S9: Functional group F1 scores for a model trained on only IR with the new definitions of functional groups

Functional Group	Training set F1	Validation set F1
Alkane	0.983110	0.935748
Alkene	0.951914	0.825343
Alkyne	0.966157	0.869274
Alcohols	0.985552	0.935951
Amines	0.969121	0.873207
Nitriles	0.887506	0.598101
Aromatics	0.997007	0.981913
Alkyl halides	0.966182	0.865727
Esters	0.970721	0.912860
Ketones	0.965129	0.867477
Aldehydes	0.979790	0.903850
Carboxylic acids	0.977540	0.930756
Acyl halides	0.945896	0.788083
Amides	0.832065	0.595560
Methyls	0.977781	0.932062
Ethers	0.984980	0.923053
Nitros	0.990951	0.931536

Table S10: Functional group F1 scores for a model trained on FTIR and MS spectra with the new definitions of functional groups

	Molecular Perfection Rate	Molecular F-1
**Training set**	79.1323%	0.955077
**Validation set**	64.0335%	0.909212

Table S11: MPR and MF1 values for a multitask model trained on only FTIR spectra with the new definitions of functional groups

	Molecular Perfection Rate	Molecular F-1
**Training set**	87.8871%	0.975642
**Validation set**	65.2510%	0.912017

Table S12: MPR and MF1 values for a multitask model trained on FTIR and MS spectra

Functional Group	Training set F1	Validation set F1
Alkane	0.968777	0.93169
Alkene	0.907346	0.812864
Alkyne	0.945042	0.851205
Alcohols	0.97892	0.944236
Amines	0.946405	0.852841
Nitriles	0.717182	0.488428
Aromatics	0.992644	0.974879
Alkyl halides	0.907742	0.810426
Esters	0.979923	0.922709
Ketones	0.951888	0.867387
Aldehydes	0.976048	0.918015
Carboxylic acids	0.971139	0.941297
Acyl halides	0.920298	0.791876
Amides	0.788451	0.597016
Methysl	0.963815	0.932059
Ethers	0.973455	0.923417
Nitros	0.98336	0.946973

Table S13: Functional group F1 scores for a model trained using an autoencoder on only FTIR with the new definitions of functional groups

Functional Group	Training set F1	Validation set F1
Alkane	0.984136	0.932257
Alkene	0.947195	0.819603
Alkyne	0.958650	0.848086
Alcohols	0.978334	0.910960
Amines	0.960173	0.852991
Nitriles	0.854644	0.553305
Aromatics	0.996893	0.982649
Alkyl halides	0.963728	0.855594
Esters	0.969606	0.913754
Ketones	0.964384	0.857152
Aldehydes	0.979850	0.866663
Carboxylic acids	0.979510	0.917079
Acyl halides	0.952464	0.736802
Amides	0.844232	0.557778
Methyls	0.978014	0.930977
Ethers	0.980859	0.919104
Nitros	0.987188	0.933380

Table S14: Functional group F1 scores for a model trained using an autoencoder on FTIR and MS with the new definitions of functional groups

	Molecular Perfection Rate	Molecular F-1
**Training set**	78.8955%	0.955907
**Validation set**	62.5593%	0.904820

Table S15: MPR and MF1 values for a model trained using an autoencoder on only FTIR and with the new definitions of functional groups

	Molecular Perfection Rate	Molecular F-1
**Training set**	86.8895%	0.973454
**Validation set**	62.5726%	0.905013

Table S16: MPR and MF1 values for a model trained using an autoencoder on FTIR and MS and with the new definitions of functional groups

References

[1] S. Kim, P. A. Thiessen, E. E. Bolton, J. Chen, G. Fu, A. Gindulyte, L. Han, J. He, S. He, B. A. Shoemaker, et al., *Nucleic Acids Res.* **2016**, *44*, DOI 10.1093/nar/gkv951.

[2] G. A. Landrum, **n.d.**

[3] DayLight, **n.d.**

[4] R. Nalla, R. Pinge, M. Narwaria, B. Chaudhury, in *Proc. ACM India Jt. Int. Conf. Data Sci. Manag. Data - CoDS-COMAD '18*, **2018**, pp. 201–209.

[5] R. J. Fessenden, L. Györgyi, *J. Chem. Soc., Perkin Trans. 2* **1991**, 1755–1762.

[6] E. W. Robb, M. E. Munk, *Mikrochim. Acta* **1990**, *100*, 131–155.

[7] F. Chollet, Keras, **2015**.

[8] J. Gabel, J. Desaphy, D. Rognan, *J. Chem. Inf. Model.* **2014**, *54*, 2807–2815.

[9] J. T. Springenberg, A. Dosovitskiy, T. Brox, M. Riedmiller, **2014**.

[10] M. D. Zeiler, R. Fergus, in *Lect. Notes Comput. Sci. (Including Subser. Lect. Notes Artif. Intell. Lect. Notes Bioinformatics)*, **2014**, pp. 818–833.
